# Improving cardiovascular population risk in primary care: protocol for the PROSPERA cluster-randomised controlled trial of a complex multilevel intervention

**DOI:** 10.1136/bmjopen-2026-118002

**Published:** 2026-10-27

**Authors:** Vera A M C Bongaerts, Rimke C Vos, Steven H J Hageman, Ken Redekop, Jannick A N Dorresteijn, Mattijs E Numans, Hendrikus J A Van Os

**Affiliations:** 1 Public Health and Primary Care, Leiden University Medical Center, Leiden, Netherlands; 2 Vascular Medicine and Endocrinology, University Medical Centre Utrecht, Utrecht, Netherlands; 3 Erasmus School of Health Policy and Management, Erasmus University Rotterdam, Rotterdam, Netherlands; 4 Psychiatry, University Medical Centre Utrecht, Utrecht, Netherlands

**Keywords:** Cardiovascular Disease, Primary Health Care, Randomized Controlled Trial, Risk Assessment, Clinical Decision-Making, Patient-Centered Care

## Abstract

**Introduction:**

The growing burden of cardiovascular disease (CVD) threatens the sustainability of our healthcare systems. In the Netherlands, two-thirds of CVD-related healthcare is delivered in primary care practices. Given the increasing staffing shortages and the substantial heterogeneity of the population of primary care patients at increased risk of CVD, cardiovascular risk management strategies should be better tailored to individual patients’ needs and risk profiles. Therefore, we developed the PROSPERA programme, a population-level risk-based approach aimed at improving cardiovascular risk management in primary care.

**Methods and analysis:**

The PROSPERA study is a pragmatic 1:1 cluster randomised controlled trial in 22 primary care practices in the greater Leiden and The Hague region in the Netherlands, investigating real-world effectiveness of usual care plus the PROSPERA programme with usual care alone. The PROSPERA programme consists of (1) a population-level risk stratification overview to anticipate care needs (empanelment), (2) training for healthcare professionals in cardiovascular risk communication, (3) a lifestyle questionnaire completed by patients before consultation (Lifestylecheck) and (4) a clinical decision support tool (U-Prevent). The programme aims to support a more targeted use of available resources by identifying patients at higher risk and tailoring preventive efforts to those who may benefit most, thereby potentially improving the efficiency and effectiveness of care. Patients between 40 and 90 years of age enrolled in integrated care programmes (programmes developed to ensure a continuum of care for patients with chronic conditions) for increased cardiovascular risk are included. The primary outcome is defined as the proportion of the participants with successful achievement of all protocol-defined cardiovascular preventive treatment goals at 18 months of follow-up. Secondary outcomes include implementation outcomes (penetration and fidelity), healthcare professionals’ satisfaction on usability of the PROSPERA programme, patient experience with shared decision-making and decisional conflict when making a healthcare decision and outcomes on cost-effectiveness.

**Ethics and dissemination:**

Consent for the use of routine healthcare data are obtained through an opt-out procedure for participating practices. Digital informed consent will be obtained for the study questionnaires for patients and healthcare professionals. Main findings on effectivity, efficacy and implementation will be disseminated via peer-reviewed journals and via (inter)national conferences.

**Trial registration number:**

NCT06593704.

**STRENGTHS AND LIMITATIONS OF THIS STUDY**

- The PROSPERA programme was co-developed with stakeholders and refined via a preceding qualitative study, ensuring better integration with clinical workflows.
- The pragmatic design of this study allows for broad inclusion at the level of the population at increased risk of cardiovascular diseases, enhancing representativeness and inclusivity.
- Use of the Extramural LUMC Academic Network (ELAN) data infrastructure enables comprehensive collection of healthcare data.
- Potential cross-over contamination cannot be prevented, as control practices may have informal access to some PROSPERA components, although these are not yet widely implemented.

## Introduction

 Cardiovascular diseases (CVD) are the leading cause of death for people worldwide.^1^ The growing disease burden and the associated costs endanger the sustainability of our healthcare systems.^2^ Therefore, improving prevention of CVD is key, since most of the risk is caused by modifiable risk factors.^3^ In clinical practice, cardiovascular risk management (CVRM) is usually addressed at the level of the individual but is generally insufficient at a population level. This is supported by the wide variation in percentages of CVD risk factor control across the cardiovascular population.^4–6^

In the Netherlands, about two-thirds of the patients at risk of CVD are treated in primary care practices by a general practitioner (GP) or a practice nurse (PN) in integrated care programmes.^4^ These are programmes developed to ensure a continuum of care for patients with chronic conditions such as increased risk of CVD or diabetes mellitus (DM)—a population with substantial heterogeneity regarding individual cardiovascular risk and patient needs.^7^ However, the base frequency of CVD consultations in integrated care programmes is currently not determined by the magnitude of a patients’ cardiovascular risk, and there is at present no structure in place to identify patients for prioritised outreach. Hence, present CVD integrated care programmes can largely be considered a ‘one-size-fits-all’ approach, while tailoring cardiovascular prevention to the needs of the individual patient may be crucial to ensure CVRM stays accessible and in compliance with current guidelines.^8 9^

In recent years, advances in availability of routine care data and cardiovascular risk prediction models have enabled automated individual risk prediction. This has led to development of clinical decision support tools for individual risk predictions.^10^ However, for healthcare professionals in primary care, the use of these support tools is still suboptimal.^11 12^ Considering both this limited uptake of support tools at the individual level and the previously mentioned absence of risk stratification instruments that provide a population-level overview, a logical next step would be developing a multilevel programme that addresses both domains simultaneously.

While previous studies have made efforts to move in this direction by developing a model with age-specific and sex-specific risk thresholds prioritising individuals for CVD risk assessment,^13^ an integrated multilevel approach remains undeveloped. Such an approach could enable prioritising preventive care strategies to those who most need it, while simultaneously ensuring compliance with guidelines and accommodating individual-specific needs. We therefore developed the PROSPERA programme, a multilevel complex intervention addressing both the population level and the level of the individual patient.^14^ At a population level, the PROSPERA programme provides the healthcare professional with the opportunity to proactively direct available healthcare resources based on differing risk levels in the CVD population, differentiating between who is most in need of intensive treatment versus who may require less frequent consultations. It also includes a healthcare professional training programme in cardiovascular risk communication. At the individual level, the PROSPERA programme consists of (1) an individual-level Lifestylecheck questionnaire, and (2) an individual-level clinical decision support tool (U-Prevent). The rationale underlying the programme is its potential to assist healthcare professionals in their decision-making concerning patient prioritisation for consultation and the scheduling of follow-up appointments. Although the current Dutch CVRM guideline allows flexibility in this regard, most PNs lack concrete guidance to support their decision-making. We therefore hypothesise that using risk stratification on a population level as well as risk communication tools during consultation will (1) optimise which patients to invite in what frequency for the healthcare professional and (2) when invited a patient, provide better insight into personal risk, motivation and activation threshold for the patient.

The pragmatic PROSPERA cluster randomised controlled trial (cRCT) follows a preceding qualitative study,^14 15^ and aims to improve the cardiovascular risk profile for the population of patients enrolled in integrated care programmes for increased cardiovascular risk in primary care. Secondary outcomes include implementation outcomes (fidelity and penetration), healthcare professionals’ satisfaction on usability of the PROSPERA programme, patient experience on shared decision-making and decisional conflict and cost-effectiveness.

## Methods and analysis

This study protocol is reported in accordance with the Standard Protocol Items: Recommendations for Intervention Trials (SPIRIT) reporting guidelines.

### Study design

The PROSPERA cRCT follows a pragmatic hybrid type II effectiveness and implementation study to address both effectiveness and implementation outcomes simultaneously within one study.^16 17^ For the definition of the present study as a hybrid type II study, we followed the framework by Curran *et al*.^17 18^ According to this framework, hybrid studies exist on a continuum and the distinction of a study between hybrid type I or hybrid type II is determined by the nature of the study outcomes and the extent to which implementation strategies are used and evaluated. As our study has a dual focus formulating research questions about both the effectiveness of the intervention and its implementation strategies, the hybrid design of PROSPERA is classified as a type II study.

The PROSPERA cRCT will be conducted in primary care practices affiliated with the Extramural Leiden University Medical Center Academic Network (ELAN) in the greater Leiden and The Hague region, the Netherlands, containing approximately 150 primary care practices. Primary care practices using a compatible electronic health record (EHR) will be recruited via ELAN, as well as through newsletters distributed by regional GP organisations and via posters displayed at the GP out-of-hours services for GPs on call. Primary care practices will receive a fixed financial compensation for participating. Figure 1 shows an anticipated randomisation infographic. Clustered randomisation to either the intervention (PROSPERA programme) or control group (care-as-usual) will be performed in a 1:1 ratio on the level of primary care practices, through Castor Electronic Data Capture via a validated variable block randomisation model.^19^ The anticipated number of eligible patients will depend on the size of the participating primary care practices. Allocation will only be visible for the researcher after inclusion and randomisation of the primary care practice. Blinding of primary healthcare professionals and patients will not be possible due to the nature of the intervention. The anticipated start date for inclusion was set for February 2025. Confirmed patient inclusion started in April 2025. The study is expected to span a total duration of 18 months (anticipated end date October 2026).

**Figure 1:**
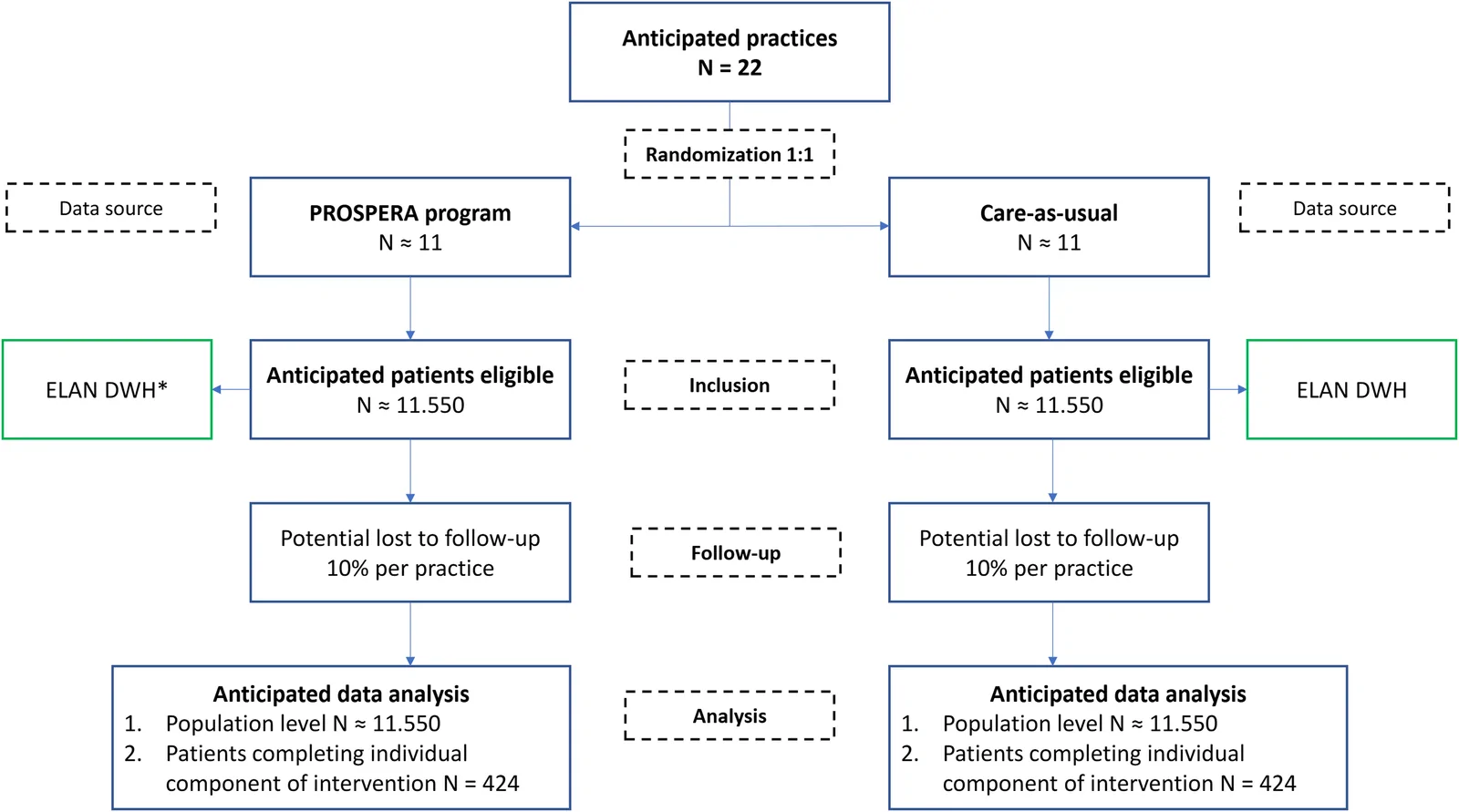
Anticipated randomisation infographic. Infographic showing the participant flow through the study, projected recruitment of practices, randomisation to intervention and control arms, data source and estimated number of eligible patients, anticipated loss to follow-up and expected number of patients for the final analyses. *ELAN DWH, Extramural Leiden University Medical Center Academic Network Data Warehouse.

### Study population

Patients are eligible for inclusion if they meet all the following criteria: age ≥40 years and ≤90 years, enlisted with one of the participating primary care practices and enrolled in one of the Dutch integrated care programmes for (increased risk of) CVD or DM. As this study aims to establish real-world effectiveness of the PROSPERA programme, we aligned with current guidelines for patients participating in existing integrated care programmes. Within these integrated care programmes, individuals between 40 years and 90 years of age can be enrolled. This also reflects the age range for which the risk calculators are validated. Patients will be excluded if they receive palliative care, are pregnant, are pre-dialysis or in a kidney transplantation procedure, have a form of cognitive impairment, are treated in other integrated care programmes because of their frailty (eg, elderly who are treated in the ‘Frail Elderly’ integrated care programme) or if they are diagnosed with DM type I.

### Care-as-usual

The comparator in this study will be standard cardiovascular risk management provided by primary care healthcare professionals in integrated care programmes for (increased risk of) CVD or DM. This care-as-usual means that patients with an increased risk for cardiovascular disease receive annual invitations for CVRM consultations to ensure continuity of care in accordance with the Dutch Cardiovascular Risk Management Guidelines of General Practitioners.^9 20^ There is currently no patient prioritisation structure in place. Within these programmes, the GP—and not the specialist in the hospital—must provide CVRM and patient motivation is required for enrolment. PNs are often the professionals that execute the consultations within the integrated care programmes under GP supervision. PNs independently provide primary care to specific groups of patients with chronic diseases. They focus on prevention, monitoring, patient education and the provision of support and guidance to patients.^21^ PNs are generally employed by individual GPs or general practice groups. Before qualifying, practice nurses complete a 1-year or 2-year education programme. PNs assume responsibilities from the GP; however, the GP retains final responsibility. In the PROSPERA study, the healthcare professionals who routinely provide CVRM care-as-usual in their primary care practice will also be responsible for reviewing the empanelment lists every 3 months, prioritising patients for invitation and the consultations with patients using the Lifestylecheck and U-Prevent. This process may be conducted by either a GP or a PN. A prerequisite for using the empanelment list to prioritise patients and conducting individual PROSPERA CVRM consultations is completion of the PROSPERA accredited training in cardiovascular risk communication and participation in the initiation visit.

### Intervention

The PROSPERA programme constitutes a complex intervention, and its development, implementation and evaluation will be conducted according to the UK Medical Research Council (MRC) framework for developing and evaluating complex interventions and follows the four phases for complex intervention research: (1) development of the intervention (2) feasibility testing (3) evaluation and (4) implementation.^14^ For this reason, the development and feasibility of the PROSPERA programme and its accompanying information technology structures were tested in a qualitative study before initiating this subsequent cRCT.^15 22 23^ As a result of insights gained from this qualitative study, minor adaptations were made to the PROSPERA programme and supporting implementation strategies were put into practice.^24^ An accredited training programme for healthcare professionals was added as a population intervention component to support cardiovascular risk communication. In addition, identified implementation and behavioural change strategies will be put into practice. An implementation ambassador will be appointed in each primary care practice participating in the intervention arm. This GP or PN serves as the primary point of contact for the researchers and is offered implementation strategies (eg, training, user guides, pocket cards) for guiding the implementation of the intervention. At the start of the trial, we will conduct an initiation visit in each primary care practice in order to familiarise the healthcare professionals with the PROSPERA programme. During this visit, we will systematically work through all components and all stages of the programme, including all supporting materials.

Other implementation strategies incorporated into the study include aligning the intervention with specific and frequently used EHRs, stimulating healthcare professionals’ engagement with the programme by distributing newsletters with tips and tricks, organising Q&A sessions and improving the accessibility of the intervention by restructuring the digital and physical environment in the consultation room (ie, providing intervention components such as the Lifestylecheck in paper format or by placing a desktop shortcut to the automatically pre-filled U-Prevent medical device on the computer) during the initiation visit.^24^ Implementation strategies will be evaluated via the pre-defined implementation outcomes and during a final implementation gap visit at the end of the study.

Figure 2 shows a schematic representation of the intervention components of the PROSPERA programme. The PROSPERA programme consists of interconnected population-level and individual-level components designed to deliver proactive and targeted CVRM. The programme will start with a risk stratification and prioritised outreach at a population level for eligible patients. It follows with an accredited training for healthcare professionals to improve cardiovascular risk communication in the consultation room. At the individual level, the programme continues within a subset of the population invited for consultation with personal prevention for the individual patient using U-Prevent and the Lifestylecheck.^10 25^ Figure 3 shows a user flow of the PROSPERA programme for healthcare professional and patient.

**Figure 2:**
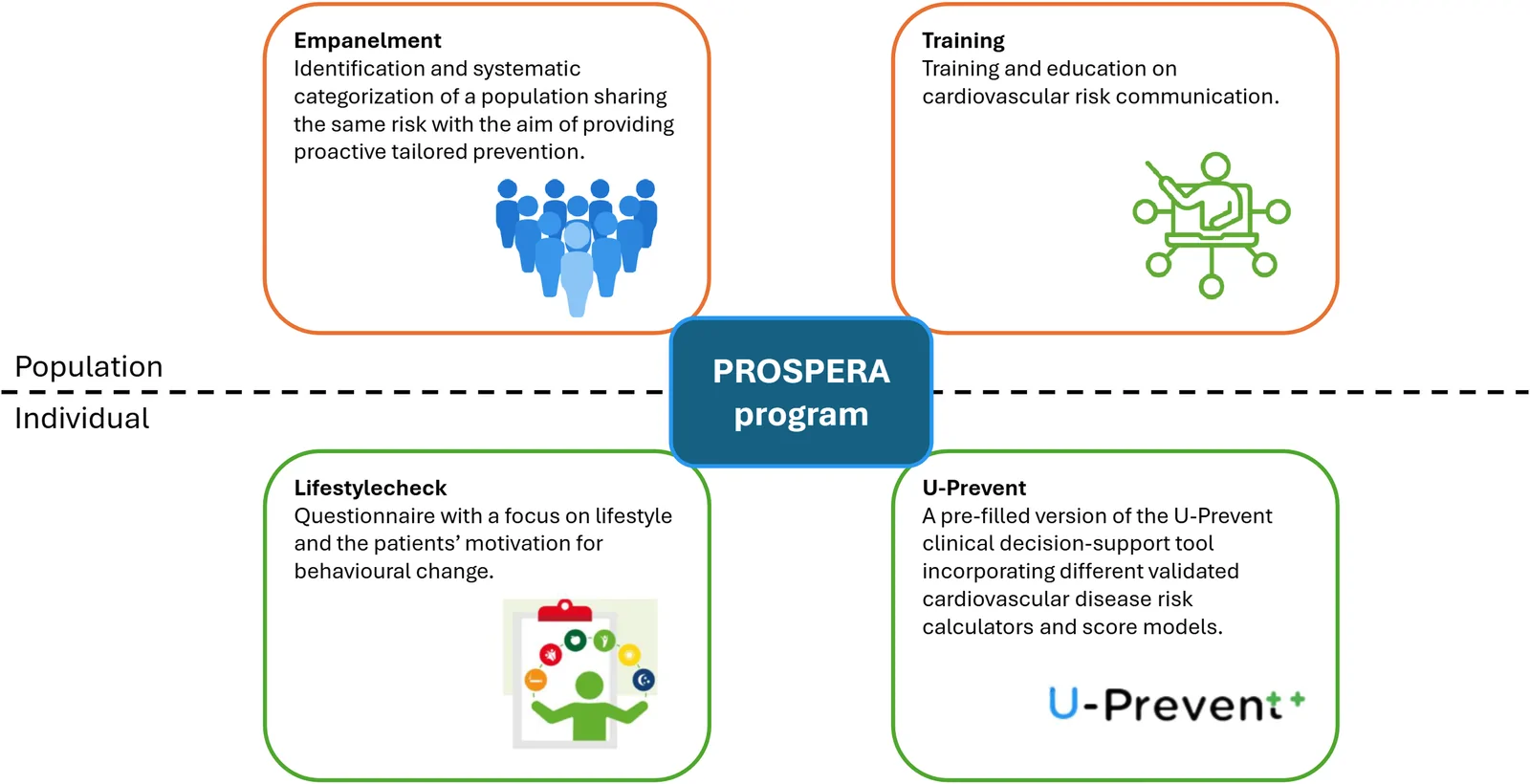
Components of the PROSPERA programme. Schematic representation of the PROSPERA programme illustrating intervention components at both the population and the individual levels.

**Figure 3:**
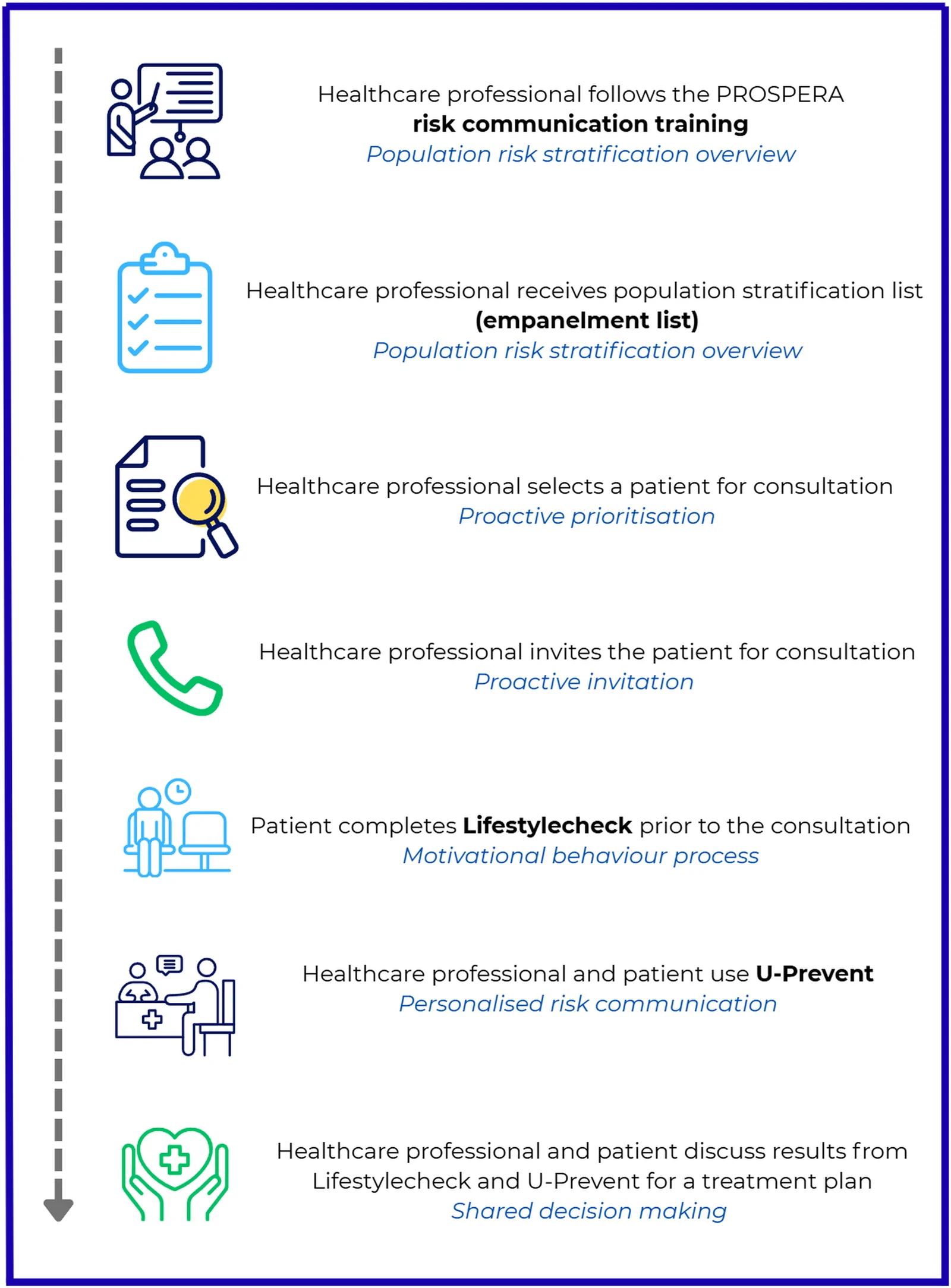
PROSPERA user flow for healthcare professional and patient. Flow diagram illustrating the pathway from cardiovascular risk communication training for healthcare professionals to using the individual PROSPERA components in clinical practice.

#### Empanelment

At a population level, PROSPERA applies a population-level risk stratification categorising patients into three panels. This process is referred to as empanelment. Empanelment generally adopts the idea that after initial segmentation of the target population, further risk stratification allows healthcare professionals to proactively direct healthcare that aligns with the needs and health conditions of the respective individual.^26 27^

In PROSPERA, empanelment is used as an analytical stratification tool. It is a recurrent process in which routinely collected clinical data from EHRs, such as medical history, blood pressure measurements and laboratory results, are used to stratify patients at risk of CVD according to their anticipated care needs. The resulting stratification supports healthcare professionals in the organisation and proactive delivery of primary care based on anticipated care demand, rather than reactively providing care once symptoms of progression emerge. Figure 4 shows a schematic representation of the empanelment process in PROSPERA.

**Figure 4:**
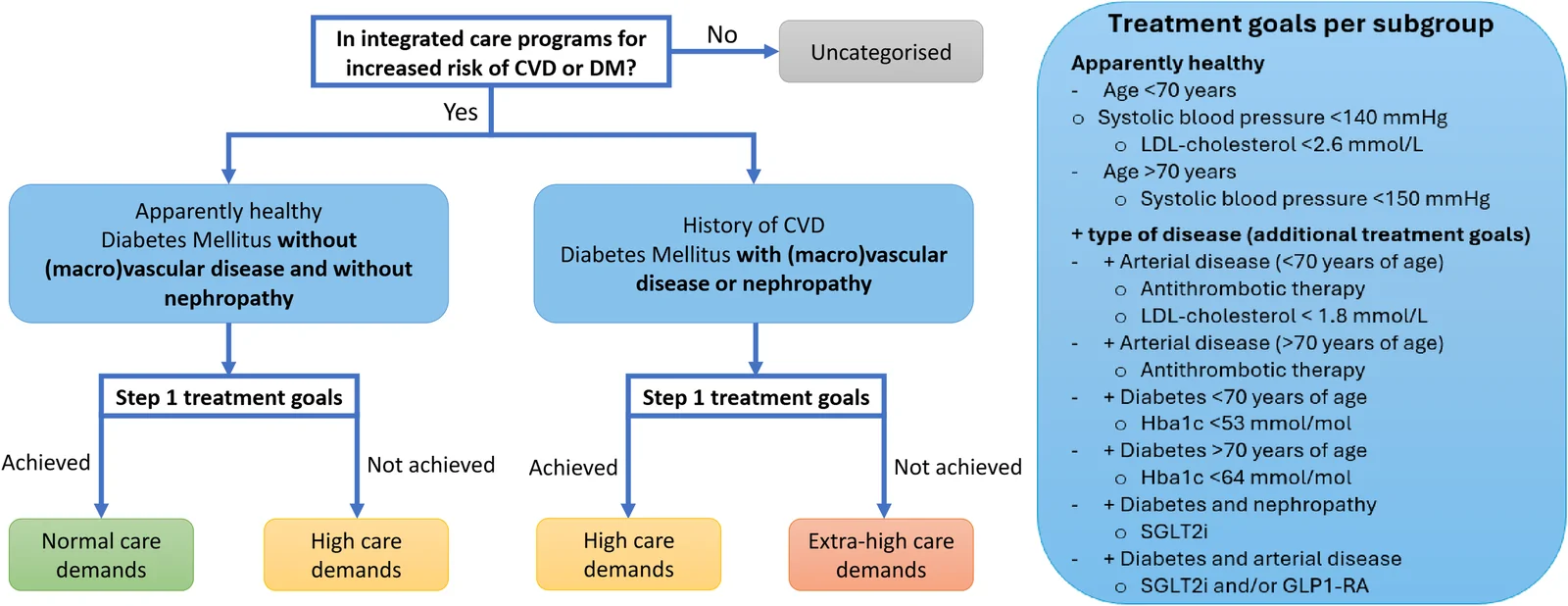
Patient empanelment and protocol defined treatment goals per subgroup. Flow diagram showing empanelment of patients enrolled in integrated care programmes according to primary or secondary cardiovascular prevention status and achievement of predefined treatment targets. Treatment goals per subgroup are indicated. CVD, cardiovascular disease; DM, diabetes mellitus.; GLP-1 RA, glucagon-like peptide-1 receptor agonist; HbA1c, glycated haemoglobin; LDL, low-density lipoprotein; SGLT2i, sodium-glucose cotransporter-2 inhibitor.

Creation of the PROSPERA panels is based on two criteria. First, whether the patient requires primary (apparently healthy) or secondary (history of CVD) cardiovascular prevention. Second, whether the patient has achieved the predetermined preventive CVD treatment goals based on the European Society of Cardiology (ESC) Clinical Practical Guidelines, the Dutch Cardiovascular Risk Management Guidelines of General Practitioners and the Dutch Diabetes Mellitus type 2 Guidelines of General Practitioners.^8 9 28^ This leads to three PROSPERA panels: normal, high or extra-high. Further details on the panels and treatment goals are outlined in online supplemental appendix A. Panels are updated every 3 months. The first empanelment marks the starting point of the PROSPERA cRCT (T=0).

Missing clinical data for patients in integrated care programmes, such as missing blood pressure measurements, missing low-density lipoprotein (LDL)-cholesterol levels or missing glycated hemoglobin (HbA1c) levels for patients with DM, that are missing for >2 years, will be considered as treatment goals not achieved, given that individuals enrolled in integrated care programmes are invited on a regular basis for risk factor control. We assume that enrolled patients who have missing clinical data will have either somehow disengaged from follow-up or otherwise have been overlooked by the healthcare system. We have chosen to consider them as treatment goals not achieved in the empanelment procedure to alert the healthcare professional.

#### Empanelment implications

Empanelment is implemented as a population-level tool for proactive care management and is conducted by the research team every 3 months. Participating primary care practices receive an overview of their integrated care population at risk for CVD, stratified according to expected CVRM care demand into three panels: normal, high and extra-high. During the training and the initiation visit, the research team will guide healthcare professionals in interpreting and appropriately using this overview.

The purpose of the empanelment overview is to enable healthcare professionals to identify patients who might benefit from proactive CVRM management and to prioritise patients for invitation and follow-up. Thus, panel assignment does not constitute a treatment algorithm and does not prescribe a specific clinical intervention. Rather, it provides structured information to support decisions about which patients to prioritise, when to invite them for CVRM review and how intensively to follow them.

Although the decision as to how to act on the population risk stratification remains with the healthcare professional, the intended implications of the three normal, high and extra-high panels were as follows. Patients in the normal panel generally required less proactive CVRM management when treatment goals were achieved and no relevant CVD history was present. These patients could therefore be reviewed at a lower frequency, with renewed CVRM assessment according to the usual clinical context and changes in health status. Patients in the high panel could be prioritised for more active CVRM management because of an increased need for risk-factor assessment and/or treatment optimisation. For these patients, healthcare professionals could use the individual-level PROSPERA components, including U-Prevent and the Lifestylecheck to support individual risk assessment, lifestyle assessment and shared decision-making. Patients in the extra-high panel, characterised by a history of CVD and treatment goals not being achieved, could be prioritised for the most intensive proactive management and follow-up, with the aim of identifying unmet treatment needs and supporting treatment optimisation.

Population empanelment for proactive prevention is new in Dutch primary care. Our previous qualitative research showed that healthcare professionals use it as an addition to their own judgement on who to invite for consultation first.^24^

#### Risk communication training

Healthcare professionals in the intervention group will attend a mandatory 2-hour in-person accredited training session on cardiovascular risk communication. The training will be organised in collaboration with an educational institute in the Netherlands specialised in education on the subject of cardiometabolic diseases for healthcare professionals in primary care.^29^ Learning objectives include the translation of cardiovascular risk and benefit calculations from U-Prevent to understandable information for the patient. The training will cover topics like the concept of lifetime risk, 10-year risk, CVD-free life expectancy and number needed to treat. Trainers will provide examples on how to translate these percentages and numbers to understandable information for the patient. The training will include practice with fictional patient cases. Healthcare professionals will be provided with the opportunity to review the recorded training online and attend 1-hour question-and-answer sessions two times online with the trainers.

#### Lifestylecheck

The Lifestylecheck is a questionnaire dedicated to promoting healthier lifestyle behaviours in the Dutch population.^25^ In the PROSPERA programme the Lifestylecheck will be adopted as a pragmatic tool to discuss current lifestyle and behavioural change incentives for eligible patients with an increased risk of CVD. The patient is asked to complete the questionnaire as preparation before consultation. Since patients indicate their motivation for behavioural change on the questionnaire, it can be used by the healthcare professional to guide motivated shared decision-making in combination with the U-Prevent clinical decision support tool.^25 30^

#### U-Prevent

The U-Prevent medical device is used as a clinical decision support tool during consultation between healthcare professional and patient. U-Prevent incorporates different validated CVD risk calculators and score models in one digital application.^10 31 32^ Models used in U-Prevent to predict 10-year cardiovascular risk are SCORE2, SCORE2-OP, SCORE2-Diabetes and SMART2. The SMART-REACH, DIAL2 and LIFE-CVD2 score models are used for lifetime risk and treatment-modelling predictions. The device complies with the transitional requirements of the Medical Device Regulation (EU 2017/745). Additional information on the functionalities, intended use and user manuals of U-Prevent can be accessed via the website (www.u-prevent.com). The use of U-Prevent is recommended in the 2021 ESC guidelines on cardiovascular disease prevention in clinical practice.^8^ These guidelines describe the role of risk prediction via U-Prevent in current evidence-based cardiovascular preventive care. The use of U-Prevent is also endorsed in Dutch General Practice Standards for Cardiovascular Risk Management and U-Prevent is currently used on a small scale by healthcare professionals working in both primary and secondary care in the Netherlands.^9 20 31^ The version of U-Prevent used in this study is adapted for study purposes by pre-filling available relevant risk predictors such as biographical information, clinical measurements, laboratory findings and current medication use from the electronic health records to ease the workflow of the healthcare professional. U-Prevent and the Lifestylecheck are discussed during consultation between healthcare professional and patient to prioritise treatment goals. See figure 3 for the PROSPERA user flow for both healthcare professional and patient.

### Data management and infrastructure

Data will be obtained via questionnaires and via ELAN. ELAN is a dynamic data infrastructure originating from clinical-academic collaboration and supported by quarterly extracted pseudonymised routine healthcare data from affiliated organisations such as hospitals and primary care practices.^33^ Data from ELAN is only accessible on request and with permission from the ELAN committee. In the PROSPERA study this routine healthcare data will be used for the empanelment, the pre-filling of U-Prevent and for assessing the primary and secondary outcomes. It includes, among others, coded clinical measurements, laboratory findings, information on medication use and information on smoking behaviour.^33 34^ Identifiable patient data are pseudonymised for researchers and other stakeholders, but accessible for healthcare professionals via a trusted third party in the Netherlands responsible for data processing (see figure 5).^35^ A Data Management Plan is in place at dmponline.lumc.nl and accessible on request.

**Figure 5:**
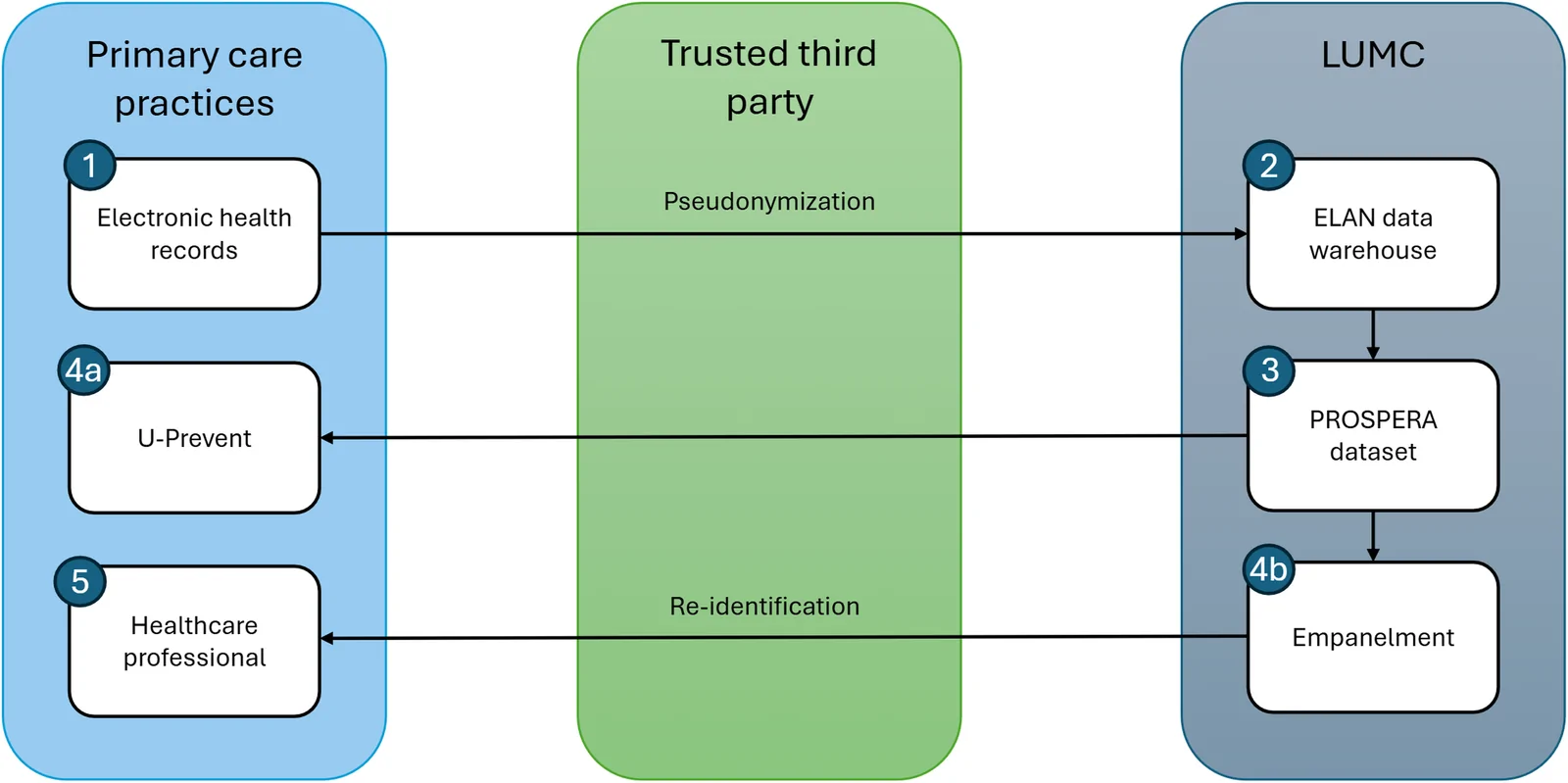
PROSPERA data infrastructure. Steps: (1) Routine healthcare data are extracted from electronic health records in primary care practices. Data are pseudonymised via a trusted third party. (2) Data are stored in ELAN data warehouse. (3) Data are requested for research. (4a) Data are used to pre-fill in U-Prevent. (4b) Data are used for PROSPERA empanelment. (5) Healthcare professionals have access to re-identifiable data to be used in clinical practice. ELAN, Extramural Leiden University Medical Center Academic Network; LUMC, Leiden University Medical Center.

### Primary outcome

The primary outcome is defined as successful achievement of all protocol-defined cardiovascular preventive treatment goals, measured as the difference between the proportion of patients who meet the outcome in the intervention versus the control group at 18 months.

In addition to the primary population analysis, we will perform a prespecified subanalysis across all included practices for patients in the intervention group who received all components of the PROSPERA programme.

### Secondary outcomes

Secondary outcomes include (1) the implementation measures fidelity and penetration, (2) outcomes on healthcare professionals’ satisfaction on usability of the PROSPERA programme, (3) outcomes on patient experience with shared decision-making and decisional conflict and (4) outcomes on cost-effectiveness. Secondary patient outcomes will only be assessed for a percentage of the patients within the intervention arm receiving all intervention components. Table 1 shows the specifications per questionnaire.

**Table 1:** Specifications per questionnaire

Patient	Questionnaire	MM and time duration	Number of items	Range	Interpretation
Cost-effectiveness	EQ-5D-5L	MM=after the intervention and after 12 months. Time: 2 min.	6	5-point scale (converted to a 0–100 range)	Low scores indicate the best health state, high scores indicate health problems
Shared decision-making	Shared Decision-Making Questionnaire	MM=after the intervention. Time: 3 min.	9	6-point scale (converted to a 0–100 scale)	Higher scores indicate a greater extent of shared decision-making
Decisional conflict	Decisional Conflict Scale*	MM=after the intervention. Time: 3 min.	16	3-point scale (converted to a 0–100 range)	Higher scores indicate more decisional conflict
Healthcare professional	**Questionnaire(s**)	**Estimated time**	**Number of items**	**Range**	**Interpretation**
Fidelity	PROSPERA Fidelity Scale	MM=at month 4 and 10. Time: 5 min.	16	0–100 scale	Higher scores indicate higher fidelity and adherence
Usability	System Usability Scale	MM=at month 10. Time: 2 min.	10	5-point scale (converted to a 0–100 range)	Higher scores indicate higher usability

* The low literacy version translated to Dutch.

MM, measurement moment.

#### Implementation outcomes

Fidelity is the extent to which the PROSPERA programme components are used as intended. Fidelity will be assessed in two ways: (1) through a PROSPERA Fidelity Scale for primary healthcare professionals designed for the purpose of this study (see online supplemental appendix B), and (2) as the percentage of the target patient population that has received the U-Prevent component of the PROSPERA programme during the study duration, as measured by the U-Prevent logbook. Higher scores indicate higher fidelity and adherence to the study protocol. Penetration is calculated as the number of healthcare professionals who deliver the PROSPERA programme on an individual level, divided by the number of healthcare professionals who are expected to deliver this service. Implementation outcomes will be classified according to the taxonomy as proposed by Proctor *et al*.^36^

Implementation outcomes will be used to better understand whether variation in fidelity and penetration across practices is related to clinical effectiveness variation.^17 18^ We will explore whether the effectiveness of the intervention, as measured by the primary outcome at the individual practice level, is associated with the extent to which the intervention was delivered as intended and which implementation strategies were used. These exploratory associations will not be interpreted causally, but are intended to explain heterogeneity in the observed outcomes. The evaluation of the implementation determinants and used strategies will also inform development of further implementation strategies aimed at facilitating the transition of the PROSPERA intervention from trial-based research setting to routine clinical practice in the future.

As this study is a pragmatic RCT, the substantial heterogeneity in the organisational structure of primary care practices in the Netherlands makes it difficult to anticipate all contextual factors a priori. Therefore, the PROSPERA programme is standardised across intervention practices, while allowing for local adaptations during the trial period. Such variation is not considered a protocol deviation, provided that the programme’s general principle of providing proactive and tailored care to meet the true needs of an individual patient is maintained.^37^ Fidelity to the programme will be monitored through bi-monthly contact with the intervention ambassadors, during implementation gap visits and quantitively using the fidelity scale completed two times during the study by the healthcare professionals to assess whether the intervention is used as intended.

#### Healthcare professional outcomes

Healthcare professionals’ satisfaction on usability of the PROSPERA programme will be measured for healthcare professionals in the intervention arm, by using the validated System Usability Scale, where higher scores indicate higher usability.^38^ See table 1 for measurement moments.

#### Patient outcomes

Perceived shared decision-making and decisional conflict will be measured in a subset of 15% of patients registered at practices in the intervention arm, selected by the healthcare professional within a predefined period of time. Healthcare professionals will start after patient 15 and continue until they have included six patients per practice for the patient questionnaires. As inviting patients to complete questionnaires proved to be a time-consuming barrier for the healthcare professionals following our preceding qualitative study, we opted to limit this to this subset in order to minimise the burden.^24^ Shared decision-making will be assessed using the validated 9-item Shared Decision Making Questionnaire for patients.^39^ Higher scores indicate a greater extent of shared decision-making. Decisional conflict will be assessed using the validated Decisional Conflict Scale for low literacy.^40^ Higher scores indicate more decisional conflict.

### Cost-effectiveness

The cost effectiveness of the PROSPERA programme in primary care practices in the Netherlands will be assessed using a cost-utility analysis with quality adjusted life years and life-years as the two primary effectiveness measures. A lifetime time horizon and a societal perspective will be applied. The trial results will be combined with other data sources to estimate the long-term costs and effects of PROSPERA. Besides multiple one-way sensitivity, subgroup and scenario analyses, a probabilistic sensitivity analysis will be conducted to quantify the uncertainty surrounding the incremental costs, effectiveness and incremental cost-effectiveness ratio. A cost-effectiveness acceptability curve will show the likelihood that PROSPERA is cost effective at different willingness-to-pay thresholds. Lastly, a budget impact analysis (BIA) for implementation of the PROSPERA programme in the Netherlands (minimum period, 3 years) will be performed based on data collected via ELAN and other sources. Both the cost-effectiveness analysis (CEA) and BIA will be performed according to the Dutch guidelines for economic evaluations in healthcare.^41^

### Sample size calculation

Since all enrolled patients will receive the population-level intervention components (empanelment and healthcare professional training), but only a subset will receive the additional individual-level components, we have prespecified a subanalysis of the primary outcome within this subset. Since the clinical and implementation importance of this subanalysis, we chose to determine the sample size based on the required minimum number of patients expected to complete all components of the PROSPERA programme. The study is powered to find a clinically relevant difference of 10% between the care-as-usual subset and the PROSPERA subset in reaching all protocol-defined cardiovascular preventive treatment goals. In the Netherlands, the standard practice size per full time equivalent GP is 2095 patients.^42^ On average, two to three GPs work together in a single primary care practice. Based on insights from literature and findings from our previously conducted qualitative study, we conservatively estimated that approximately 350 patients per one full time equivalent GP will meet the inclusion criteria.^4^ A power of 80% and an alpha of 5% were chosen as the statistical parameters for the sample size rationale. Assuming an intraclass correlation coefficient of 0.05 based on previous literature,^43–45^ this resulted in an estimated sample size of 11 clusters (primary care practices) with 35 patients per cluster and a total of 385 patients per study arm. Consulted healthcare professionals indicated that a number of 40 patients per primary care practice would be feasible to treat following all components of the PROSPERA programme during the course of the study. Accounted for a potential loss-to-follow-up of approximately 10% (eg, due to patients moving to a different region or discontinuing the integrated care programme) the total sample size amounts to 848 patients over 22 primary care practices (with a true sample size of 770 patients), with 424 patients (with a true sample size of 385 patients) in the intervention arm receiving the individual-level components of the PROSPERA programme.

### Statistical analysis

The primary outcome will be analysed following the intention-to-treat principle. A mixed model is developed to determine the proportion of patients who have achieved all protocol-defined cardiovascular preventive treatment goals in the intervention group compared with the control group at the end of the study versus at the start. The start of the study was defined as the moment of PROSPERA empanelment. Data to assess the primary outcome will be extracted from ELAN using a dataset with an extraction date closest after the end of the follow-up period. Bias from measurement errors is expected to be randomly distributed across intervention and control practices since routine healthcare data are used to assess the outcomes of this study. Categorical data will be presented as numbers and percentages. Continuous variables will be summarised using means and SDs for approximately normally distributed data, and medians with IQRs for skewed distributions.

Subgroup analyses will be performed for age and sex. We will also conduct a within patient comparison between pre-PROSPERA and post-PROSPERA programme for included patients who received all components of the PROSPERA programme. These outcomes will be analysed using multivariable logistic regression, adjusted for confounders, with GP practice included as random effect to account for clustering of patients within practices. A p value of 5% will be considered clinically significant.

Since missing data on relevant CVD prevention treatment goals may move participating patients into a higher panel, we anticipate that this could partially affect the primary outcome. To account for this, a sensitivity analysis will be performed on multiple imputed treatment indicators.

The implementation outcomes, fidelity and penetration and the questionnaires will be analysed using descriptive statistics. For the assessment of fidelity, we will use user data on adherence to the U-Prevent component of the PROSPERA programme. Categorical data will be presented as numbers and percentages. Continuous variables will be presented as means with SDs or median and IQR, as appropriate.

#### Exploratory analyses

In our exploratory analyses we will also examine the difference between the intervention and control group in (1) the net improvement of PROSPERA panel distribution, (2) the number of patients (%) that remained stable in the normal risk panel over the course of the study, (3) the improvement in other clinical patient-related outcomes between pre-implementation and post-implementation of the intervention between baseline and follow-up, that is, in terms of risk score (%), LDL (absolute reduction in mmol/L) and systolic blood pressure (absolute reduction in mm Hg), (4) the proportion (%) of patients with complete risk factor data registered between baseline and end of follow-up, (5) the absolute number (or %) of patients with (re)admission to the cardiovascular or DM integrated care programmes between baseline and end of follow-up and (6) the difference between high-socioeconomic and low-socioeconomic position practices in reaching the primary outcome.

### Clinical implications

The PROSPERA programme may have an impact on both the primary care practice and the individual patient. At the level of primary care practices, a standardised risk-based structure for prioritised outreach and subsequent follow-up of the population of patients at risk of CVD is currently absent in the Netherlands. Primary care practices use a wide variety of approaches and invite patients opportunistically or reactively, based on surname, date of birth or the date of their previous visit. In a healthcare climate where scarcity is becoming ever more prominent, the PROSPERA programme proposes an alternative proactive risk-based population approach for patients at risk of CVD in primary care. We expect the programme to provide support to GPs and PNs in prioritising high-risk cardiovascular patients, that is, the patients who need urgent attention or intensification of preventive care. Simultaneously, it has the potential to provide a structured approach to reducing follow-up frequency for low-risk cardiovascular patients, who could instead be supported by remote patient monitoring or increased self-management. Insights from this study regarding the proactive population approach to CVRM of the PROSPERA programme could be extended to other countries in which primary care is structured similarly with the GP as gatekeeper, such as the UK, Canada, Australia, New Zealand and Scandinavian countries. However, since complex interventions such as the PROSPERA programme are context-dependent, we assume that in countries where a risk-based outreach is already in place, the potential for achieving additional impact in terms of equity becomes smaller and research into factors that hinder or facilitate sustainable implementation should be conducted.

At the individual level, the PROSPERA programme is expected to result in improvement of traditional clinical risk predictors such as a decrease in LDL-cholesterol or systolic blood pressure through intensive counselling as part of the PROSPERA programme, with a focus on tailored prevention and a basis in enhanced risk communication in combination with the visualisations of the patients’ individual treatment effect and cardiovascular-free life expectancy in U-Prevent. Similarly, we expect the visualisations and the Lifestylecheck to contribute to the process of shared decision-making and decrease in decisional conflict.^46^ Therefore, even without a statistically significant improvement in achievement of traditional clinical risk factors in individual patients, the current trial may lead to valuable insights in how the different components of PROSPERA may interact. These insights may explain how PROSPERA might be meaningful in improving behavioural—and lifestyle factors through the emphasis on internal—and external motivation of a patient, personal drive and the increased attention to contextual factors that play a role in lifestyle change or treatment adherence necessary for the prevention of CVD. According to recent guidance of the MRC, such insights in inner mechanics of complex interventions are necessary for the improvement of development—and implementation of such interventions.^14^

### Ethical considerations and dissemination

This study will be conducted according to the principles of the Declaration of Helsinki (October 2024) and in accordance with the General Data Protection Regulation (V.OJ L 119, 04.05.2016; cor. OJ L 127, 23.5.2018). The Medical Ethical Review Board of Leiden University Medical Center mandated the review of research not falling under the Dutch Medical Research with Human Subjects Law (nWMO) to individual nWMO committees at Leiden University Medical Center. The nWMO committee reviewed the study proposal and provided a declaration of no objection (non-WMO approval number: 23–3050, 5 June 2023). A protocol amendment was written for this cRCT. None of the changes rendered the study subject to the Dutch Medical Research with Human Subjects Law. This study has been further reviewed and approved by the scientific committee of the department of Public Health and Primary Care at Leiden University Medical Center (WSC approval number 2023–13, 28 June 2024).

Consent for the use of routine healthcare data were obtained through an opt-out procedure for participating practices. For the study questionnaires listed in table 1 we will obtain digital informed consent via Castor EDC. Questionnaires will also be distributed to the healthcare professionals and patients via Castor EDC. The trial is registered at http://www.clinicaltrials.gov under registration number NCT06593704 (5 September 2024). The trial is currently enrolling by invitation. Results on effectivity, efficacy and implementation will be disseminated via peer-reviewed journals and via (inter)national conferences to the scientific community and the public. Participating primary care practices and other stakeholders will receive update newsletters during the study about its progression and will be informed per email about the results. Results are expected in 2028.

## Supplementary material

10.1136/bmjopen-2026-118002 online supplemental file 1

10.1136/bmjopen-2026-118002 online supplemental file 2
