# Supplementary material for: Improving cardiovascular population risk in primary care: protocol for the PROSPERA cluster-randomised controlled trial of a complex multilevel intervention
Source: BMJ Open. 2026 Oct 27;16(10):e118002. doi: 10.1136/bmjopen-2026-118002 (PMC13723831; doi:10.1136/bmjopen-2026-118002)
Supplement: Supplementary file 3 [file bmjopen-2026-118002.reviewer_comments.pdf]

# PEER REVIEW HISTORY

BMJ Open publishes all reviews undertaken for accepted manuscripts. Reviewers are asked to complete a checklist review form and are provided with free text boxes to elaborate on their assessment. These free text comments are reproduced below.

## ARTICLE DETAILS

**Title (Provisional)**

Improving cardiovascular population risk in primary care: protocol for the PROSPERA cluster-randomized controlled trial of a complex multilevel intervention

**Authors**

Bongaerts, Vera A. M. C.; Vos, Rimke C; Hageman, Steven HJ H.J.; Redekop, Ken; Dorresteijn, Jannick A.N.; Numans, Mattijs E.; van Os, Hendrikus J A

---

## VERSION 1 - REVIEW

---

| | |
|---|---|
| **Reviewer** | **1** |
| **Name** | **Hespe, Charlotte** |
| **Affiliation** | **The University of Notre Dame Australia, School of Medicine** |
| **Date** | **07-Apr-2026** |
| **COI** | **None** |

---

This protocol describes an ambitious and potentially important cluster randomised trial of a multilevel cardiovascular prevention intervention in primary care in the Netherlands. The overall rationale is strong, and the alignment with MRC guidance for complex interventions and SPIRIT reporting is evident. However, there are currently substantial gaps in clarity and internal consistency that limit my ability to understand what is actually being delivered in practice, how patients move through the intervention, how population-level and patient-level components relate to one another, and how the preceding qualitative work informed the final study design.

For a protocol paper, these issues are particularly important, as transparency, reproducibility, and interpretability are core purposes of protocol publication. Substantial clarification is therefore required before the study design and intervention can be fully appraised.

The key issues requiring clarification are outlined below.

1. Sample size, recruitment flow, and internal consistency of numbers

The manuscript states that each practice can feasibly enrol approximately 35 patients, leading to:

11 practices × 35 patients = 385 patients per arm

770 patients total

The authors then state that, allowing for 10% loss to follow-up, the total sample increases to 848 (or 847) patients, with 424 per arm, but there is no explanation of how these additional patients are recruited or from where they arise. This is difficult to reconcile with the earlier statement regarding feasibility limits at the practice level. A clear explanation of the recruitment strategy, attrition assumptions, and how sample size targets will be achieved in practice is required. A flow diagram or table mapping recruitment, inclusion, and analysis populations would greatly improve clarity.

2. Insufficient description of empanelment and the patient journey

It is not clear whether empanelment is primarily an analytic stratification tool or whether it mandates specific clinical actions. The protocol does not adequately explain:

what it means in practice for a patient to be categorised as "normal", "high", or "extra-high" risk;

what differential care, intensity, frequency, or additional support is expected for patients in each category; or

how clinicians are expected to act on panel assignment beyond receiving an education module.

Similarly, it is unclear whether U-Prevent is completed by patients independently prior to consultation, during consultation with a healthcare professional, or via a combination of approaches.

Overall, the protocol does not yet provide a sufficiently concrete description of what happens to an individual patient in the intervention arm.

3. Blurring of population-level and patient-level intervention components

The distinction between population-level and patient-level components requires clearer conceptualisation. For example, the clinician education module is described as an individual-level intervention, yet it is delivered at clinician or practice level and affects all patients. Empanelment applies to the entire eligible population, while primary analyses focus on a patient subgroup receiving additional components. Clear definitions and justification for how intervention components are categorised and analytically handled are needed.

4. Insufficient support for claims regarding prior qualitative work

The manuscript states that a preceding qualitative study informed intervention development. However, the cited references describe methodological frameworks rather than presenting findings from the authors' qualitative work. The protocol should explicitly

describe the qualitative study undertaken (methods, participants, and key insights) or provide an appropriate citation if this work has been published.

5. Unclear differentiation of care or support across risk panels

Related to points 2 and 3, there is no explicit description of how care differs between patients classified as normal, high, or extra-high care demand. Without clarification of whether and how intervention intensity varies, it is difficult to interpret both clinical and implementation outcomes.

6. Appendix B provided only in Dutch

The PROSPERA Fidelity Scale (Appendix B) is central to the assessment of implementation outcomes but is presented only in Dutch. An English translation should be provided to enable meaningful appraisal by international readers.

7. Role of U-Prevent insufficiently described

A clearer, plain-language explanation of U-Prevent's role in the consultation (e.g. risk calculator, decision aid, behaviour-change support tool, or combination thereof) would enhance understanding. An appendix description would be appropriate.

8. Handling of missing data requires further justification

The protocol states that missing routine data will be automatically classified as "treatment goals not achieved", but does not adequately acknowledge or discuss the potential for systematic bias introduced by this approach.

9. Relationship between implementation fidelity and clinical effectiveness outcomes

While the protocol appropriately includes both implementation and effectiveness outcomes, the relationship between these domains is insufficiently articulated. It is unclear how variation in fidelity across practices will be considered when interpreting clinical outcomes, particularly if effectiveness is modest or heterogeneous. Clarification of how implementation findings will be used to contextualise or explain effectiveness results would strengthen analytic coherence.

Thank you for submitting this protocol paper and I wish you well in the implementation of this study.

---


| | |
|---|---|
| **Reviewer** | **2** |
| **Name** | **Halcomb, Elizabeth J.** |
| **Affiliation** | **University of Wollongong Faculty of Science Medicine and Health, School of Nursing** |
| **Date** | **10-Apr-2026** |
| **COI** | **None** |


---

This is a well written proocol paper for an interesting study that has the potential to inform future policy and practice. While the introduction sets the scene for this study, it could touch more on other international studies that have a similar approach to demonstrate learnings from them. Similarly a little more detail on the following points would help ensure that the reader shares the authors understnadings;

a) justification of this very broad age inclusion criteria

b) for an international audience defining the term “practice nurse” and explining this in the study context

c) some comment about the nature of the adaptations after the qual study would help the reader understand what was done

d) a little more detail and clarity about the specific roles of the GP and PN and collaboration

---

| | |
|---|---|
| **Reviewer** | **3** |
| **Name** | **Ridgeway, Jennifer** |
| **Affiliation** | **Mayo Clinic, Center for the Science of Health Care Delivery** |
| **Date** | **11-Apr-2026** |
| **COI** | **None** |

---

Thank you for the opportunity to review this study protocol paper. The authors nicely portray their overall study design. Readers would benefit from more details in a several areas, though, including the introduction, description of usual care and intervention components, and the rationale and specification of implementation outcomes. Detailed comments are listed below.

Abstract

Description of the intervention as having three patient-level components might better be described as having three individual-level components, as most are aimed at changing clinician behavior.

Strengths and limitations

Even after reading the paper, the statement “The complexity of the current information- and technology (IT) infrastructure prototype is a constraint, but is expected to be simplified in the future” is unclear to me. Furthermore, if there is a key component that is expected to change, the authors should address any implication such changes will have on their study.

Introduction

The argument for which care gaps are being addressed by the intervention (starting at Line 35, Page 4) could be better articulated. This would also help the reader understand why the team has selected the outcomes of interest. This includes clearer description of how the

intervention addresses staff shortages, for example, and how it helps better integrate CDS tools in the consultation room. Likewise, the argument for why the population approach alleviates clinician burden (rather than adding more work to clinicians who need to review and make decisions) would be helpful.

Methods

• Study design: Rationale for a hybrid type 1 study is needed, e.g., the reason for including implementation outcomes and the selection of a type 1 design rather than another design.

• Study design: More detail on site selection would help readers understand how the 22 practices will be identified and recruited to participate. Likewise, information about incentives and potential for drop-out would are needed.

• Study population: Rationale for ages 40-90 is needed.

• Care as usual and intervention description: As noted above, the description of the intervention and the figure would be more helpful if it was clear what mechanism and outcome they target. More details on components would also help readers, including more details on the training (e.g., content and duration), as well as how they fit together. Portraying an example of the process for a hypothetical clinician and patient (in the text of a figure) may help readers tie these pieces together better. Finally, clearer comparison of the intervention vs usual care components would also help readers, for example in a table.

• Primary outcome: I find Figure 5 to be difficult to follow. The figure lacks footnotes to help make sense of abbreviations, but it also does include traditional timeline information.

• Secondary outcomes: The secondary outcomes could be better specified and aligned with the intervention components. Details about how, when, and with whom (intervention and control?) surveys are conducted are needed, as well as rationale for including only 15% of patients for SDM questionnaire and rationale for random selection by clinicians.

• Implementation outcomes: There is very little detail and no rationale for the implementation outcomes selected. I was unable to assess the fidelity scale, and the description is insufficient to understand what is being assessed (fidelity to using components, delivering SDM and risk communication, attending training, completing questionnaires) and why, i.e., what will the team do with fidelity data? Likewise, how exactly is penetration measured and how will those findings be used? Similar limitations are found in the analysis section.

• Adaptation and context: The authors state: “Therefore, adaptation to the context of a specific primary care practice is not directly seen as a deviation from the intervention as long as the general principle of the PROSPERA program, namely providing proactive and tailored care to meet the true needs of an individual patient, was not lost in the process.” While I appreciate the need for adaptation to context, this statement raises questions about how context will be assessed and adaptations will be identified. Furthermore, it seems to blur the line between practice-level adaptations and individualized patient-centered care.

Other considerations

• I do not see approval or registration dates or the status of enrollment.

---

## VERSION 1 - AUTHOR RESPONSE

## Response to the reviewers' comments

***Reviewer #1***

**General remarks of the reviewer**
*This protocol describes an ambitious and potentially important cluster randomised trial of a multilevel cardiovascular prevention intervention in primary care in the Netherlands. The overall rationale is strong, and the alignment with MRC guidance for complex interventions and SPIRIT reporting is evident. However, there are currently substantial gaps in clarity and internal consistency that limit my ability to understand what is actually being delivered in practice, how patients move through the intervention, how population-level and patient-level components relate to one another, and how the preceding qualitative work informed the final study design. For a protocol paper, these issues are particularly important, as transparency, reproducibility, and interpretability are core purposes of protocol publication. Substantial clarification is therefore required before the study design and intervention can be fully appraised.*

> **General response of the author**
> We thank the reviewer for this positive perspective and for highlighting several important points for improvement and clarification. We have considered the comments and questions raised and have addressed each point in detail below.

**Comment 1: sample size, recruitment flow, and internal consistency of numbers.**
*The manuscript states that each practice can feasibly enroll approximately 35 patients, leading to:*
*11 practices × 35 patients = 385 patients per arm*
*770 patients total*
*The authors then state that, allowing for 10% loss to follow-up, the total sample increases to 848 (or 847) patients, with 424 per arm, but there is no explanation of how these additional patients are recruited or from where they arise. This is difficult to reconcile with the earlier statement regarding feasibility limits at the practice level. A clear explanation of the recruitment strategy, attrition assumptions, and how sample size targets will be achieved in practice is required. A flow diagram or table mapping recruitment, inclusion, and analysis populations would greatly improve clarity.*

> **Authors' response**
> Patients are eligible to participate if they are enrolled in one of the Dutch integrated care programs for (increased risk of) CVD or DM.
>
> For our primary endpoint we will analyse patients in the Dutch integrated care programs receiving the population-level intervention components (population level outcomes - estimated 11.550 individuals). Besides, we will analyse the enrolled patients receiving all population- and individual components of the PROSPERA program (individual level outcomes, estimated 385-424 individuals).

The sample size of 35 patients per primary care practice derives from a power calculation for the latter. This power calculation indicates that a minimum of 35 patients per cluster (11 practices per study arm) are required to detect a clinically relevant difference of 10% between the care-as-usual group and the intervention group (the patients receiving all PROSPERA intervention components).

To account for a potential loss to follow-up of approximately 10% (e.g. due to patients moving or discontinuing the integrated care program), we inflated the recruitment target accordingly. Based on input from consulted healthcare professionals, recruitment of 40 patients per primary care practice is considered feasible. This results in a planned recruitment of approximately 39 patients per practice, which remains within the feasibility range as indicated by healthcare professionals, while at the same time providing a bit more flexibility for potential loss to follow-up.

We have relocated several sentences in the sample size section on p.14 to improve the logical flow of the argumentation.

A flow diagram mapping the recruitment and analysis is provided in the method section of the manuscript via figure 1 (see also below).

**Fig 1**. Anticipated randomization infographic

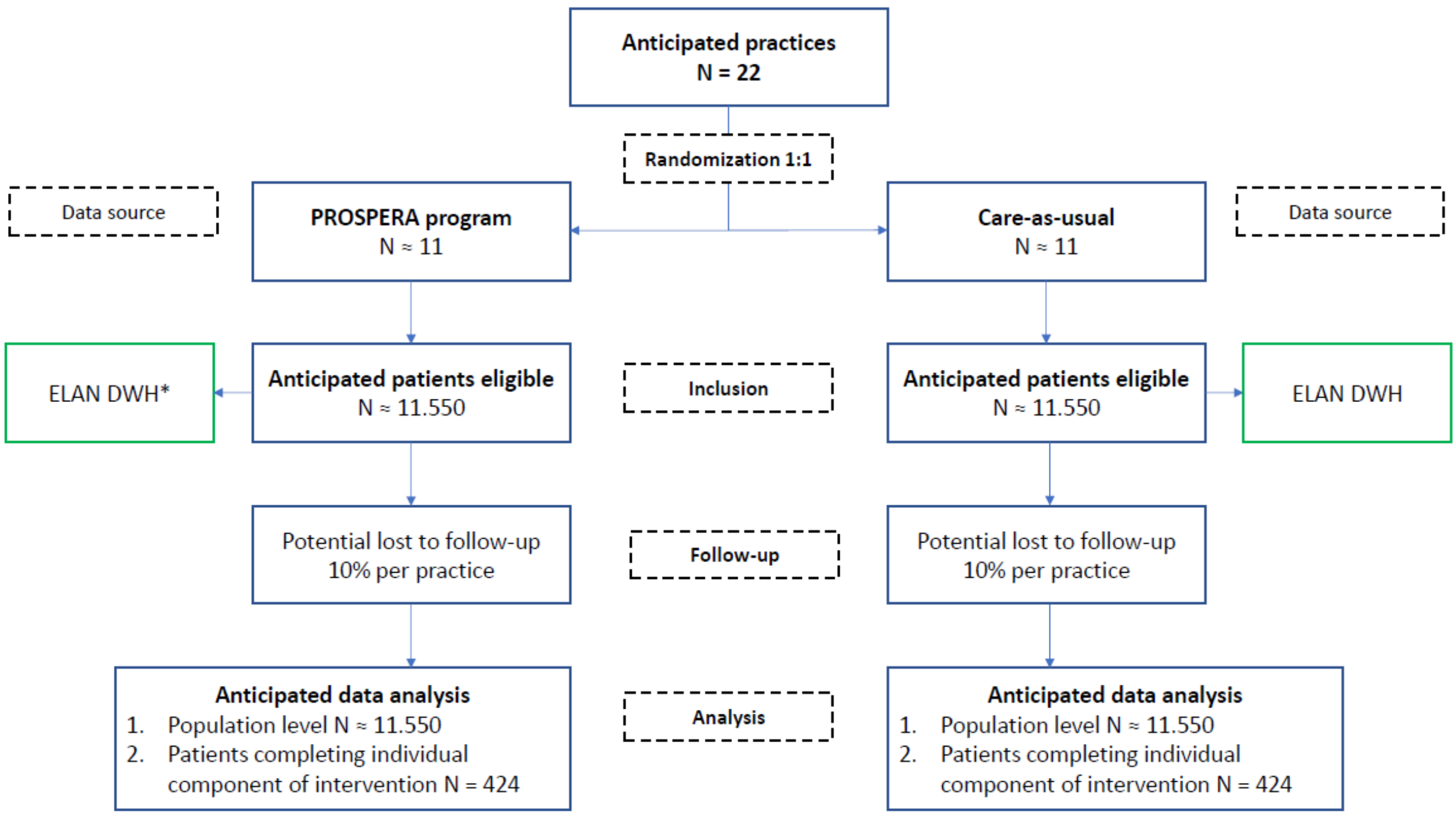


**Comment 2: insufficient description of empanelment and the patient journey.**
*It is not clear whether empanelment is primarily an analytic stratification tool or whether it mandates specific clinical actions. The protocol does not adequately explain:*
*what it means in practice for a patient to be categorised as "normal", "high", or "extra-high" risk;*
*what differential care, intensity, frequency, or additional support is expected for patients in each category; or how clinicians are expected to act on panel assignment beyond receiving an*

*education module. Similarly, it is unclear whether U-Prevent is completed by patients independently prior to consultation, during consultation with a healthcare professional, or via a combination of approaches.*
*Overall, the protocol does not yet provide a sufficiently concrete description of what happens to an individual patient in the intervention arm.*

**Authors' response**
The empanelment component of the PROSPERA program is primarily an analytic stratification tool that provides healthcare professionals with a digital overview of the integrated care population. This pragmatic support helps inform healthcare professionals in their decision making on who to prioritise for consultation, without dictating specific clinical actions. This stratified population overview for proactive prevention is new in Dutch primary care. In addition, it shows the reasons why patients are empanelled in one of the three categories, providing healthcare professionals insight both at the population level (how is the distribution across the panels) and at the individual level (why is a patient categorised in a certain risk panel). Our previous qualitative research (reference to the pre-print included in the revised manuscript) shows that healthcare professionals use it as an addition to their own judgement (1).

For the individual patient, the categorisation (normal, high, extra-high) may result in an earlier or later consultation than would otherwise occur. However, this decision is made by the HCP, the program does not mandate a specific action (such as: seeing a patient more frequent). Once a healthcare professional makes the decision to invite a patient, the individual-level components (Lifestylecheck and U-Prevent) of the intervention are initiated during consultation. The patient fills out the Lifestylecheck to indicate their motivation for behavioural change regarding unhealthy lifestyle factors. U-Prevent is prefilled with available relevant risk predictors to ease the workflow of the healthcare professional. U-Prevent and the Lifestylecheck are discussed during consultation between healthcare professional and patient to prioritize treatment goals. We have added a PROSPERA user flow (figure 4) in the manuscript to clarify this.

We have expanded the empanelment, training and U-Prevent explanation in the method section on page 8, page 9 and page 10 of the revised manuscript.

**Figure 4**. PROSPERA user flow for healthcare professional and patient

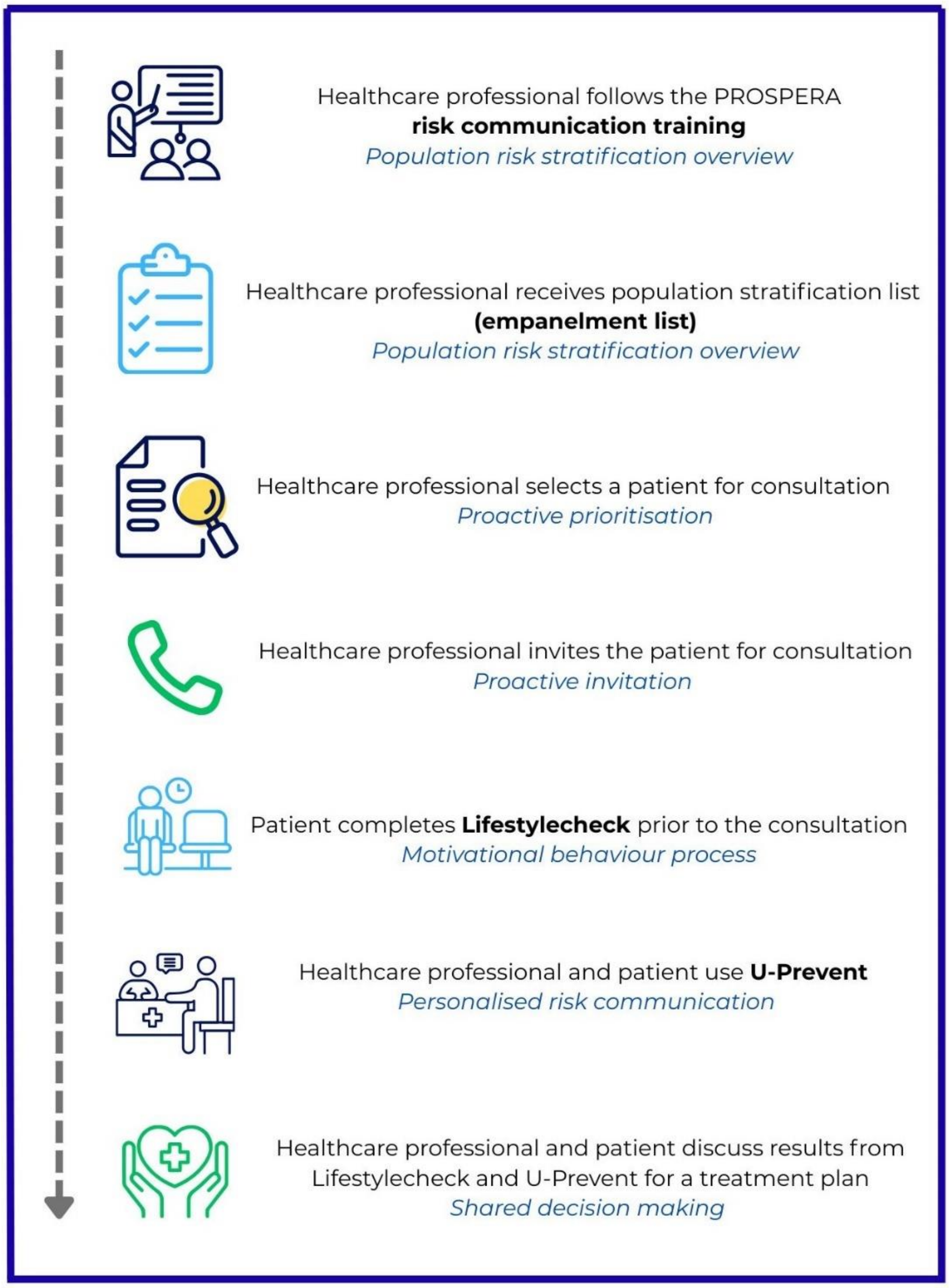


**Comment 3: blurring of population-level and patient-level intervention components.**
*The distinction between population-level and patient-level components requires clearer conceptualisation. For example, the clinician education module is described as an individual-level intervention, yet it is delivered at clinician or practice level and affects all patients. Empanelment applies to the entire eligible population, while primary analyses focus on a patient subgroup receiving additional components. Clear definitions and justification for how intervention components are categorised and analytically handled are needed.*

**Authors' response**

We thank the reviewer for raising this important comment and agree that the distinction between population level and individual level requires clarification. This issue has been carefully considered by the research team.

We agree with the reviewer that both the empanelment and the accredited training program are population-level components, while the Lifestylecheck and U-Prevent

focus on the individual. The primary analysis will be performed for the group of patients receiving the population-level components. See page 7, page 8 and figure 2 in the method section of the revised manuscript.

**Fig 2.** Components of the PROSPERA program

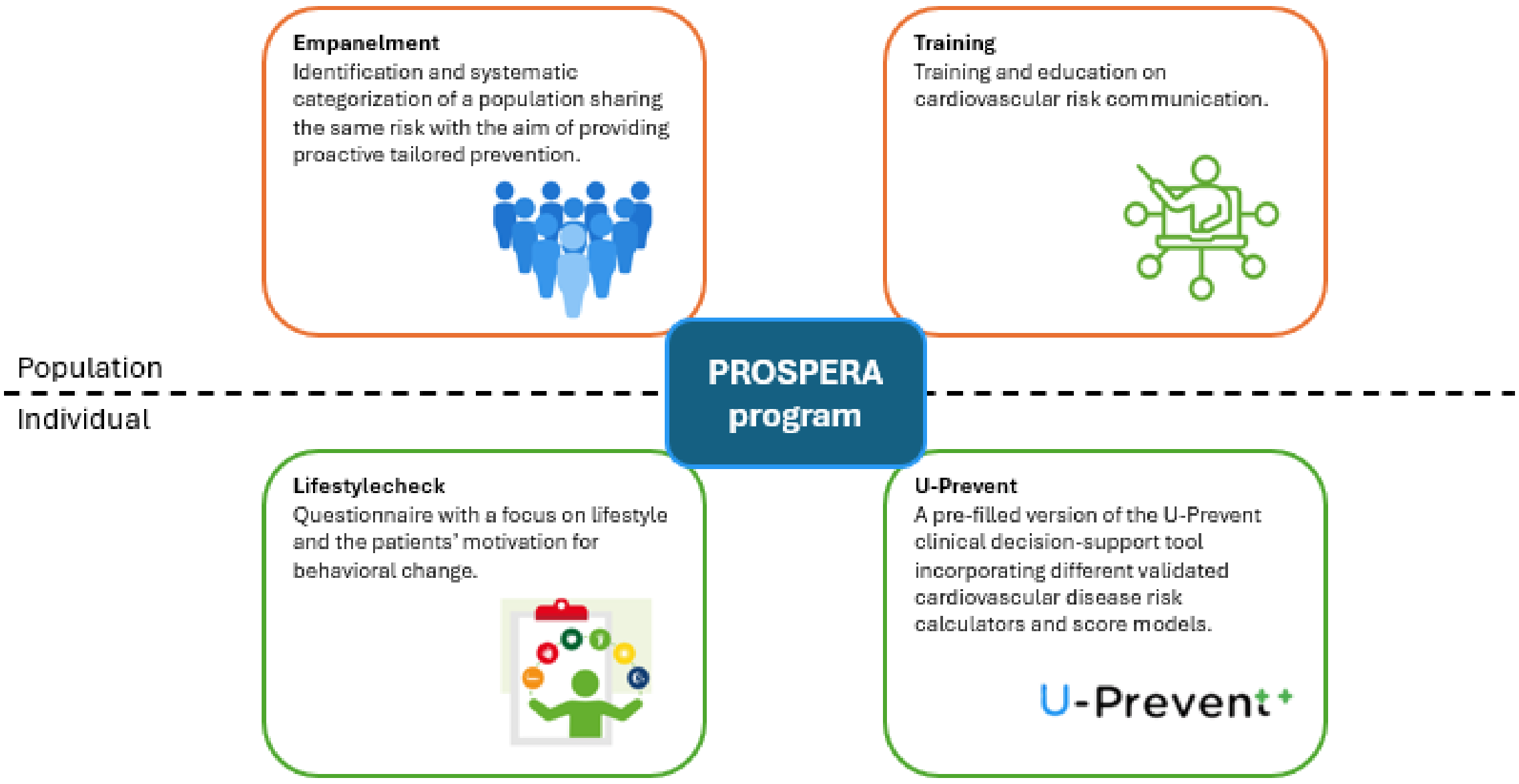


**Comment 4: insufficient support for claims regarding prior qualitative work**.
*The manuscript states that a preceding qualitative study informed intervention development. However, the cited references describe methodological frameworks rather than presenting findings from the authors' qualitative work. The protocol should explicitly describe the qualitative study undertaken (methods, participants, and key insights) or provide an appropriate citation if this work has been published.*

**Authors' response**

We have included the reference citing the preprint to the qualitative paper that informed this protocol paper and the trial. A short explanation was also added in the methodology section on p. 8, informing the reader about the changes that were made to the PROSPERA program following the preceding qualitative PROSPERA study.

'As a result of insights gained from our qualitative study, minor adaptations were made to the PROSPERA program and supporting implementation strategies were put into practice. An accredited training program for healthcare professionals was added as a population intervention component to support cardiovascular risk communication. In addition, identified implementation and behavioural change strategies such as restructuring the physical environment in the consultation room, stimulating healthcare professionals' engagement with the program and aligning the intervention with compatible EHRs, will subsequently be implemented.' (1)

**Comment 5: unclear differentiation of care or support across risk panels**
*Related to points 2 and 3, there is no explicit description of how care differs between patients classified as normal, high, or extra-high care demand. Without clarification of whether and how*

*intervention intensity varies, it is difficult to interpret both clinical and implementation outcomes.*

**Authors' response**

In responding to this comment, we would like to partially refer to our response to Comment 2.

The protocol describes a design for a pragmatic cRCT aligned with routine clinical practice, which indeed makes the selection and interpretation of outcome measures more challenging.

The intensity of the intervention, defined as the timing of progression with the individual level components of the PROSPERA program, is determined by the clinician. Healthcare professionals are not prescribed what specific actions to take.

We aim to allow healthcare professionals to tailor care to individual patients. Our intervention necessitates proactive population risk stratification combined with prioritized invitation at the clinician's discretion and a training component at the population level. At the individual level it necessitates explicit attention to effective risk communication and to assessing the patient's motivation and triggers for activation in behavior change during consultation.

This is one of the reasons why we focus on a population-level outcome measure in the context of this complex intervention. To some extent, the outcome will remain a "black box," despite our efforts to use implementation outcomes to better understand which components are adopted

**Comment 6*:* appendix B only provided in Dutch**

*The PROSPERA Fidelity Scale (Appendix B) is central to the assessment of implementation outcomes but is presented only in Dutch. An English translation should be provided to enable meaningful appraisal by international readers.*

**Authors' response:**

Thank you for your important observation. The Dutch version of the PROSPERA Fidelity Scale was inadvertently submitted. An English translation has been attached to the revised manuscript.

**Comment 7: role of U-Prevent insufficiently described.**

*A clearer, plain-language explanation of U-Prevents' role in the consultation (e.g. risk calculator, decision aid, behaviour-change support tool, or combination thereof) would enhance understanding. An appendix description would be appropriate.*

**Authors' response**

A short section on U-Prevent and its role during consultation is included on page 8 in the Method section of the manuscript:

'The U-Prevent medical device is used as a clinical decision support tool during consultation between healthcare professional and patient. U-Prevent incorporates different validated CVD risk calculators and score models in one digital application (10, 24, 25). Models used in U-Prevent to predict 10-year cardiovascular risk are SCORE2, SCORE2-OP, SCORE2-Diabetes and SMART2. The SMART-REACH, DIAL2 and LIFE-CVD2 score models are used for lifetime risk and treatment-modelling predictions.'

We have expanded this section and added a reference to the *2021 ESC Guidelines on cardiovascular disease prevention in clinical practice*. These guidelines describe the role of risk prediction in current state-of-the-art cardiovascular preventive care and the role of U-prevent for this purpose. We have also added a reference on where to access additional information on the functionalities, intended use, and user manuals of U-Prevent

**Comment 8: handling of missing data requires further justification**
*The protocol states that missing routine data will be automatically classified as "treatment goals not achieved", but does not adequately acknowledge or discuss the potential for systematic bias introduced by this approach.*

**Authors' response**
A distinction will be made between clinical judgement used in panel construction for patients with missing data and the handling of missing data in the analysis.

Individuals enrolled in integrated care programs in the Netherlands are in principle invited annually for risk factor control, unless uncontrolled risk factors warrant more visits. We assume that enrolled patients who have missing clinical data, such as blood pressure measurements or missing laboratory results, have either somehow disengaged from follow-up or otherwise have been overlooked by the healthcare system. We have chosen to consider them as treatment goals not achieved in the empanelment procedure to alert the healthcare professional. We have added this explanation in our manuscript on page 9 and 10 in the method section.

We acknowledge that this may introduce a systemic bias when analysing the results of our trial, and will address this when interpretating the results. Two lines of reasoning may be considered regarding this issue: 1) patients may already be considered sufficiently stable by the HCP and therefore are not invited for follow-up or 2) patients may avoid healthcare and monitoring, which may be associated with broader patterns of neglect.

Although the underlying reason remains uncertain, what we do know is that without the PROSPERA program, the group of patients with missing observations remains less visible.

Missing data in the analysis:
On p.16 of the manuscript there is a description on missing data in our analysis*: 'Since missing data on relevant CVD prevention treatment goals may move participating patients into a higher panel, we anticipate that this could partially affect the primary outcome. To account for this, a sensitivity analysis will be performed on multiple imputed treatment indicators.'*

**Comment 9: relationship between implementation fidelity and clinical effectiveness outcomes**
*While the protocol appropriately includes both implementation and effectiveness outcomes, the relationship between these domains is insufficiently articulated. It is unclear how variation in fidelity across practices will be considered when interpreting clinical outcomes, particularly if effectiveness is modest or heterogeneous. Clarification of how implementation findings will be used to contextualise or explain effectiveness results would strengthen analytic coherence.*

*Thank you for submitting this protocol paper and I wish you well in the implementation of this study.*

**Authors' response**

Thank you for this valuable comment. We have added a section in the manuscript on p. 13, explaining the triangulation of implementation- and effectiveness data. The implementation outcomes will be used to better understand whether variation in these outcomes is related to clinical effectiveness variation between practices. The implementation outcomes may also inform implementation strategies aimed at facilitating the transition of the PROSPERA intervention from a trial-based research setting to routine clinical practice in the future.

***Reviewer #2***

**General remarks of the reviewer**

*This is a well written protocol paper for an interesting study that has the potential to inform future policy and practice. While the introduction sets the scene for this study, it could touch more on other international studies that have a similar approach to demonstrate learnings from them. Similarly, a little more detail on the following points would help ensure that the reader shares the authors' understandings.*

**General response of the author**

While previous studies have made efforts to move in the direction of population risk stratification by developing a model with age- and sex-specific risk thresholds prioritising individuals for CVD risk assessment (Chung et al. 2023), an integrated multilevel approach remains undeveloped. Such an approach could enable prioritising preventive care strategies to those who most need it, while simultaneously ensuring compliance with guidelines and accommodating individual-specific needs.

This brief addition has been incorporated into the Introduction to better contextualise the existing evidence and gap in knowledge.

**Comment 1**

*Justification of this very broad age inclusion criteria.*

**Authors' response**

As this study aims to establish real-world effectiveness of the PROSPERA program, we aligned with current guidelines for patients participating in existing integrated care programs that ensure continuity of care for chronic conditions, such as increased risk of CVD or diabetes mellitus. Within these integrated care programs, individuals are eligible between 40 and 90 years of age. This also reflects the age range for which the risk calculators are validated. We have added this information on p. 7 in het method section.

**Comment 2**

*For an international audience defining the term "practice nurse" and explaining this in the study context.*

**Authors' response**

Practice nurses are often the professionals that execute the consultations within the integrated care programs under GP supervision. Practice nurses independently provide primary care to specific groups of patients with chronic diseases. They focus on prevention, monitoring, patient education, and the provision of support and guidance to

patients. Practice nurses are generally employed by individual GPs or general practice groups. Before qualifying, practice nurses complete a one- or two-year education program. Practice nurses assume responsibilities from the GP; however, the GP retains final responsibility.

We have included a short explanation in the method section on p.7 about the function and roles of the practice nurse in the Netherlands in our manuscript.

**Comment 3**
*Some comment about the nature of the adaptations after the qualitative study would help the reader understand what was done.*

**Authors' response**.
An accredited training program for healthcare professionals was added as a population intervention component to support cardiovascular risk communication after the qualitative study. In addition, identified implementation- and behavioral change strategies such as restructuring the physical environment in the consultation room, stimulating healthcare professionals' engagement with the program and aligning the intervention with compatible EHRs, will subsequently be implemented.

This short description is added in the methodology section on page 8, informing the reader about the changes that were made to the PROSPERA program following the qualitative PROSPERA study. We have also included the reference citing the pre-print of the qualitative paper that informed this protocol paper and the trial.

**Comment 4**
*A little more detail and clarity about the specific roles of the GP and PN and collaboration.*

**Authors' response**
See also the response on comment 2. We have provided more detail on the specific roles and collaboration of GP and PN in the Netherlands in the methods section of the manuscript on p. 8.

***Reviewer #3***

**General remarks of the reviewer**
*Thank you for the opportunity to review this study protocol paper. The authors nicely portray their overall study design. Readers would benefit from more details in several areas, though, including the introduction, description of usual care and intervention components, and the rationale and specification of implementation outcomes. Detailed comments are listed below*

**General response of the author**
We thank the reviewer for the praise and for bringing several points for improvement to our attention. We have considered the comments and have addressed each in detail below.

**Comment 1 (abstract)**
*Description of the intervention as having three patient-level components might better be described as having three individual-level components, as most are aimed at changing clinician behavior.*

**Authors' response**

We agree with the reviewer and have revised the wording from 'individual patient level' to 'individual level' throughout the manuscript. Following comment 4 made by reviewer 1, we choose to recategorize the accredited training program for healthcare professionals as a population-level component to make the distinction between population and individual clearer.

**Comment 2 (strengths and limitations)**

*Even after reading the paper, the statement "The complexity of the current information- and technology (IT) infrastructure prototype is a constraint, but is expected to be simplified in the future" is unclear to me. Furthermore, if there is a key component that is expected to change, the authors should address any implication such changes will have on their study.*

**Authors' response**

Thank you for pointing this out, we agree further clarification is necessary. In the protocol and study design for the trial, the data collection is required via the Extramural Leiden University Medical Center Academic Network (ELAN). However, direct integration between the EHR of the general practice and U-Prevent are currently being developed, enabling the pre-filled version to be accessed directly from the EHR with a single click instead of via an indirect route.

However, since this integration is not currently part of the study, we have removed this strength from the strengths and limitations section on page 3.

**Comment 3 (introduction)**

*The argument for which care gaps are being addressed by the intervention (starting at Line 35, Page 4) could be better articulated. This would also help the reader understand why the team has selected the outcomes of interest. This includes clearer description of how the intervention addresses staff shortages, for example, and how it helps better integrate CDS tools in the consultation room. Likewise, the argument for why the population approach alleviates clinician burden (rather than adding more work to clinicians who need to review and make decisions) would be helpful.*

**Authors' response**

From a population health management perspective, proactive resource allocation is only possible when a population-level overview of individual risks and missing observations is available.

The intervention itself does not directly address staff shortages, but it does provide stratification insight for healthcare professionals in the form of a digital overview of the integrated care population classified in panels.

The idea is that this could support HCPs in their decision making on who to prioritise for consultation, and in what frequency follow up should occur (Annually? Bi-annual? Once per five years?). Follow-up visits for patients may be reduced in the future, with individual-level interventions reinforcing patients' self-management of lifestyle behaviours. Although the current Dutch CVRM guideline allows flexibility in this regard, most practice nurses lack concrete guidance to support their decision-making; this is what we aim to provide.

We therefore hypothesise that using risk stratification on a population level as well as a risk communication CDS tools during consultation will a) optimize which patients to invite in what frequency for the HCP and b) when invited a patient, provide better insight into personal risk, motivation and activation threshold for him/her.

We have clarified this rationale to the introduction of the manuscript on p. 4 and 5.

**Comment 4 (methods): study design**
*Rationale for a hybrid type 1 study is needed, e.g., the reason for including implementation outcomes and the selection of a type 1 design rather than another design.*

**Authors' response**
Thank you for this attribution. We have discussed and considered this point carefully. While we originally chose to name this a hybrid type I study, we do adopt measures of implementation outcomes (fidelity, penetration) related to a hybrid type II study, and we will use certain implementation strategies from our previous qualitative work to improve uptake during this pragmatic cRCT.

We therefore concluded that our study design is more in line with a hybrid type II study than a hybrid type I study and have changed this in the revised manuscript (method section p.6, p.8 and p.13.

**Comment 5 (methods): site selection and practice recruitment**
*More detail on site selection would help readers understand how the 22 practices will be identified and recruited to participate. Likewise, information about incentives and potential for drop-out would are needed.*

**Authors' response**
Recruitment of primary care practices will be conducted through ELAN, by contacting all practices in the greater Leiden and The Hague region using a specific EHR system, through newsletters distributed by regional GP organizations and via posters displayed at the GP out-of-hours services (for GPs on call). Primary care practices will receive a fixed financial compensation for participating.

We have added additional information on recruitment in the method section on p. 6.

**Comment 6 (methods): study population**
*Rationale for ages 40-90 is needed.*

**Authors' response**
As this study aims to establish real-world effectiveness of the PROSPERA program, we aligned with current guidelines for patients participating in existing integrated care programs that ensure continuity of care for chronic conditions, such as increased risk of CVD or diabetes mellitus. Within these integrated care programs, individuals between 40 years and 90 years of age are included if eligible. This also reflects the age range for which the risk calculators are validated. We have added this information on p. 7 in het method section.

**Comment 7 (methods): care as usual and intervention description**
*As noted above, the description of the intervention and the figure would be more helpful if it was clear what mechanism and outcome they target. More details on components would also help readers, including more details on the training (e.g., content and duration), as well as how they*

*fit together. Portraying an example of the process for a hypothetical clinician and patient (in the text of a figure) may help readers tie these pieces together better. Finally, clearer comparison of the intervention vs usual care components would also help readers, for example in a table.*

**Authors' response**

In response to your comment and comment 2 of reviewer 1, we have added a user flow to the protocol manuscript, providing additional information on the mechanisms (improved doctor-patient risk communication, motivation and activation thresholds for behavioural change) instead of merely the content of the components. See figure 4 on page 5 of this document.

We have also expanded the section on the accredited training in the method section on p. 10, including more detail on the content, frequency and duration of the training and the Q&A sessions.

Besides, we have added a task description of practice nurses during care-as-usual for patients in integrated care programs in the method section on p. 6. (see also the response to comment 2 by reviewer 2).

**Comment 8 (methods): primary outcome**

*I find Figure 5 to be difficult to follow. The figure lacks footnotes to help make sense of abbreviations, but it also does include traditional timeline information.*

**Authors' response**

We have removed figure 5 from the manuscript. The time points for the questionnaires are specified in the table. In addition, the start date, end date, and study duration are now described in the method section on page 6. The figure does not add substantial additional information. However, we have added a healthcare professional and patient user flow in response to your previous comments.

**Comment 9 (methods): secondary outcomes**

*The secondary outcomes could be better specified and aligned with the intervention components. Details about how, when, and with whom (intervention and control?) surveys are conducted are needed, as well as rationale for including only 15% of patients for SDM questionnaire and rationale for random selection by clinicians.*

**Authors' response**

Thank you for your comment. We have made some additions to the secondary outcome (method) section on p.14 and p.15 in the manuscript, explaining how and with whom the surveys will be conducted. See also table 1 in the manuscript.

For the study questionnaires listed in table 1 we will obtain digital informed consent via Castor EDC. Questionnaires will be distributed to healthcare professionals and patients via Castor EDC.

Healthcare professional outcomes will be measured only for healthcare professionals in the intervention arm. The questionnaires will be distributed in months 4 and 10.

Secondary patient outcomes will be measured only in patients who have been offered the individual-level intervention components of the PROSPERA program. HCPs will start after the consultation with patient 15 and continue until they have included 6 patients per practice for the patient questionnaires. Cost effectiveness will also be measured

after 12 months. The rationale for including only 15% of patients for the patient questionnaires is that we sought to minimise the burden on healthcare professionals associated with the informed consent procedure for questionnaires, in addition to an already changing workflow. This procedure was identified as a time-consuming barrier for the healthcare professionals in our qualitative pilot study (1).

**Comment 10 (methods): implementation outcomes**
*There is very little detail and no rationale for the implementation outcomes selected. I was unable to assess the fidelity scale, and the description is insufficient to understand what is being assessed (fidelity to using components, delivering SDM and risk communication, attending training, completing questionnaires) and why, i.e., what will the team do with fidelity data? Likewise, how exactly is penetration measured and how will those findings be used? Similar limitations are found in the analysis section.*

**Authors' response**
We apologize for the inaccessible fidelity scale. A translated version has been provided with this revision. The fidelity scale assesses, among other aspects, healthcare professionals' *intention* to work with the PROSPERA program; fidelity to using the different intervention components (i.e. whether the components were delivered as is intended and, if not, how and why delivery deviated from the protocol); fidelity to enacting shared decision making; and the frequency with which (components of) the PROSPERA program were used.

Penetration is calculated as the number of healthcare professionals who deliver the PROSPERA program on an individual level, divided by the number of healthcare professionals who are expected to deliver this service (p.19 of the manuscript).

We have added a section in the manuscript explaining the triangulation of implementation- and effectiveness data. The implementation outcomes will be used to better understand whether variation in fidelity and penetration across practices is related to clinical effectiveness variation (referencing Curran et al. 2012 and 2022). The implementation outcomes and strategies may also contribute to transforming the intervention from trial-based research practice to real-life clinical practice in the future.

**Comment 11 (methods): adaptation and context**
*The authors state: "Therefore, adaptation to the context of a specific primary care practice is not directly seen as a deviation from the intervention as long as the general principle of the PROSPERA program, namely providing proactive and tailored care to meet the true needs of an individual patient, was not lost in the process." While I appreciate the need for adaptation to context, this statement raises questions about how context will be assessed and adaptations will be identified. Furthermore, it seems to blur the line between practice-level adaptations and individualized patient-centered care.*

**Authors' response**
Thank you for your words of appreciation. We will identify adaptations using the fidelity questionnaire, which includes questions for healthcare professionals regarding whether they have used the PROSPERA program as intended or which adaptations were made. We included the following information on p. 13 in the method section to make this more clear: "Adaptation to the context will be identified using the fidelity questionnaire and by regularly (once per two months) contacting the intervention practices. We will assess on

a case-by-case basis whether the study's objectives of providing proactive and tailored care are not being compromised."

**Comment 12 (other considerations)**
*I do not see approval or registration dates or the status of enrollment.*

**Authors' response**
We have added the ethical approval dates, registration dates for registration on clinicaltrials.gov. and the study's current status.

**REFERENCES**

**Reference to the pre-print of the qualitative PROSPERA manuscript:**

1. Bongaerts VAMC, van Gestel LC, van Peet PG, Vuijk M-LS, Hageman SHJ, Dorresteijn JAN, et al. Healthcare professionals' perspectives on a multilevel cardiovascular risk management intervention (PROSPERA programme). 2026.

## VERSION 2 - REVIEW

| | |
|---|---|
| **Reviewer** | **1** |
| **Name** | **Hespe, Charlotte** |
| **Affiliation** | **The University of Notre Dame Australia, School of Medicine** |
| **Date** | **14-Jul-2026** |
| **COI** | |

I appreciate the substantial work undertaken by the authors in response to the initial reviews, and I agree that the manuscript has improved in several areas. However, after reviewing the revised version, I remain concerned that several fundamental aspects of the intervention are still not described with sufficient clarity to allow a general international readership to understand exactly what the PROSPERA programme is, how it operates in practice, and how it differs from usual care. These concerns relate less to methodological rigour than to transparency, accessibility, and reproducibility of the intervention itself.

A recurring theme throughout the manuscript is that key concepts central to the intervention remain insufficiently defined or operationalised. As a result, I am still unclear about:

- what empanelment means in practical terms;

- who is actually responsible for delivering the intervention;

- what patients and clinicians experience during the intervention;

- how the intervention differs from usual cardiovascular risk management; and

- what specific changes in care result from assigning patients to different risk categories. Given that this is a protocol paper, readers should be able to understand and potentially replicate the intervention from the description provided. At present, several of these elements remain unclear.

1. Empanelment definition: Despite revisions made in response to reviewers, the concept of empanelment remains inadequately defined and continues to rely on specialised implementation science terminology. Given that empanelment is the foundational element of the PROSPERA intervention, a concise plain-language definition should be provided at first mention, together with a practical description of how the process works and how it informs clinical decision-making. This would substantially improve accessibility for an international primary care readership.

2. Accessibility of the abstract: The abstract remains difficult to follow for a broad BMJ Open readership. The language is highly technical and focuses on methodological terminology rather than clearly explaining the intervention and its intended benefits. Readers would benefit from a more plain-English description of:

- the problem being addressed;

- what the PROSPERA intervention actually involves in practice;

- how it differs from usual cardiovascular risk management; and

- why the authors believe it may improve care.

For example, terms such as "multilevel complex intervention", "population-level risk stratification", "integrated care programs", and "decisional conflict" are introduced without explanation. The abstract would be considerably strengthened if the intervention and study objectives were described in more accessible language before introducing the methodological details.

3. Definition of the intervention provider: The manuscript relies heavily on the term "healthcare professional" throughout the description of the intervention, but it remains unclear who is actually responsible for delivering the various components of the PROSPERA programme. While the roles of Dutch practice nurses are described and the manuscript notes that GPs retain overall clinical responsibility, the protocol does not clearly specify which professional groups are expected to:

- review empanelment lists,

- prioritise patient invitations,

- complete the training,

- conduct consultations using Lifestylecheck and U-Prevent,

and contribute to implementation assessments.

This is particularly important because the intervention appears to target clinician behaviour as much as patient care. A clearer description of the professional roles involved, and who is considered the primary intervention provider, would improve both understanding and reproducibility of the protocol.

4. Consequences of risk stratification: I understand how patients are assigned to risk groups, but I do not yet understand what different care those groups receive. Despite the additional explanation provided regarding empanelment, the protocol still does not clearly explain what patients in the different risk panels actually experience. The manuscript describes how patients are classified into normal, high, and extra-high care demand groups, but it remains unclear what actions are expected to follow this classification. Specifically, readers would benefit from a clear description of:

- how management differs between the three panels;

- how panel assignment influences patient invitation, review frequency, follow-up, or treatment;

- whether specific actions are recommended for each category; and

- how much of the subsequent care pathway is standardised versus left to clinician discretion.

At present, the protocol clearly describes the risk stratification process but does not clearly describe the consequences of stratification. This makes it difficult to understand the active ingredients of the intervention and how they differ from usual care.

In summary, despite your substantial revisions, several issues remain unresolved with respect to the intervention - which is insufficiently specified and inaccessible to readers.

---

| | |
|---|---|
| **Reviewer** | **3** |
| **Name** | **Ridgeway, Jennifer** |
| **Affiliation** | **Mayo Clinic, Center for the Science of Health Care Delivery** |
| **Date** | **13-Jul-2026** |
| **COI** | |

---

I thank the reviewers for their consideration of the prior comments. New details improve the transparency of the authors' work. The manuscript has remaining weaknesses, though. These are primarily related to implementation outcomes and triangulation of implementation and effectiveness data.

• The rationale for a Hybrid Type 2 study is still needed. While studying implementation and effectiveness at the same time is indeed consistent with this design, the implementation strategies and outcomes are not well specified. How are strategies implemented and

evaluated (given target implementation outcomes)? How does existing evidence inform your decision to call this a Hybrid Type 2 design?

• The description of triangulation would benefit from greater depth and explanation. How do implementation findings explain variation? What do the authors mean by triangulation in this case? How will they use fidelity data in effectiveness analyses?

• Now that I am able to assess the English version of the fidelity questionnaire, I am not convinced that it will answer questions about how the intervention was adapted for local context. As a protocol paper, this is less a design comment as a comment that this information is not clearly presented. Likewise, the procedures for assessing this on a case-by-case basis are not clear.

---

## VERSION 2 - AUTHOR RESPONSE

## Response to the reviewers' comments

**Reviewer #1**
Dr. Charlotte Hespe, The University of Notre Dame Australia

**General remarks of the reviewer**
*I appreciate the substantial work undertaken by the authors in response to the initial reviews, and I agree that the manuscript has improved in several areas. However, after reviewing the revised version, I remain concerned that several fundamental aspects of the intervention are still not described with sufficient clarity to allow a general international readership to understand exactly what the PROSPERA programme is, how it operates in practice, and how it differs from usual care. These concerns relate less to methodological rigour than to transparency, accessibility, and reproducibility of the intervention itself.*
*A recurring theme throughout the manuscript is that key concepts central to the intervention remain insufficiently defined or operationalised. As a result, I am still unclear about:*
*- what empanelment means in practical terms;*
*- who is actually responsible for delivering the intervention;*
*- what patients and clinicians experience during the intervention;*
*- how the intervention differs from usual cardiovascular risk management; and*
*- what specific changes in care result from assigning patients to different risk categories. Given that this is a protocol paper, readers should be able to understand and potentially replicate the intervention from the description provided. At present, several of these elements remain unclear.*

> **General response of the author**
> We thank the reviewer for continuing to help us improve the manuscript. We fully agree that the intervention description should be as clear as possible to ensure accessibility of the paper to its readers. Please see below the comments addressed point-by-point and the subsequent revisions we have made to the manuscript.

**Comment 1: Empanelment definition**
*Despite revisions made in response to reviewers, the concept of empanelment remains*

*inadequately defined and continues to rely on specialised implementation science terminology. Given that empanelment is the foundational element of the PROSPERA intervention, a concise plain-language definition should be provided at first mention, together with a practical description of how the process works and how it informs clinical decision-making. This would substantially improve accessibility for an international primary care readership.*

**Authors' response**

We've rewritten the paragraphs on the concept of empanelment on page 9 of the manuscript (in red) to improve clarity and accessibility. For our response to comment 1, we would also like to refer to our response to comment 4.

'At a population level, PROSPERA applies a population-level risk stratification categorising patients into three panels. This process is referred to as empanelment. Empanelment generally adopts the idea that after initial segmentation of the target population, further risk stratification allows healthcare professionals to proactively direct healthcare that aligns with the needs and health conditions of the respective individual.

In PROSPERA, empanelment is used as an analytic stratification tool. It is a recurrent process in which routinely collected clinical data from EHRs, such as medical history, blood pressure measurements and laboratory results, are used to stratify patients at risk of CVD according to their anticipated care needs. The resulting stratification supports healthcare professionals in the organisation and proactive delivery of primary care based on anticipated care demand, rather than reactively providing care once symptoms of progression emerge. Figure 3 shows a schematic representation of the empanelment process in PROSPERA.'

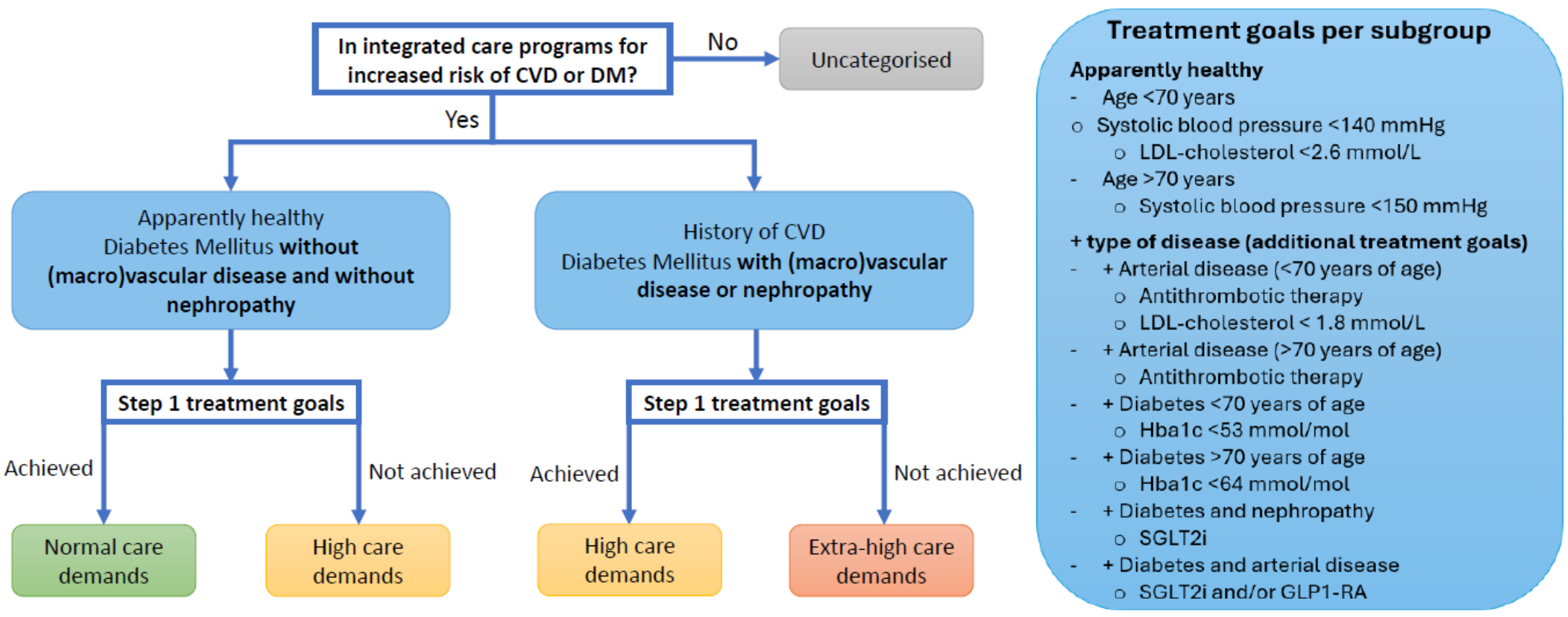


***[Figure 3]***

**Comment 2: Accessibility of the abstract**

*The abstract remains difficult to follow for a broad BMJ Open readership. The language is highly technical and focuses on methodological terminology rather than clearly explaining the intervention and its intended benefits. Readers would benefit from a more plain-English description of:*

*- the problem being addressed;*

*- what the PROSPERA intervention actually involves in practice;*

*- how it differs from usual cardiovascular risk management; and*

*- why the authors believe it may improve care.*
*For example, terms such as "multilevel complex intervention", "population-level risk stratification", "integrated care programs", and "decisional conflict" are introduced without explanation. The abstract would be considerably strengthened if the intervention and study objectives were described in more accessible language before introducing the methodological details.*

**Authors' response**
Thank you for this observation We have revised (in red) the abstract based on the suggestions above and deleted and/or explained the technical terminology, intending to improve its clarity in plain language and making it more accessible to our audience. See below in red:

**Abstract**

**Introduction** The growing burden of cardiovascular disease (CVD) threatens the sustainability of our healthcare systems. In the Netherlands, two-thirds of CVD-related healthcare is delivered in primary care practices. Given the increasing staffing shortages and the substantial heterogeneity of the population of primary care patients at increased risk of CVD, cardiovascular risk management strategies should be better tailored to individual patients' needs and risk profiles. Therefore, we developed the PROSPERA program, a population-level risk-based approach aimed at improving cardiovascular risk management in primary care.
**Methods and analysis** The PROSPERA study is a pragmatic 1:1 cluster randomized controlled trial in 22 primary care practices in the greater Leiden and the Hague region in The Netherlands, investigating real-world effectiveness of usual care plus the PROSPERA program with usual care alone. The PROSPERA program consists of 1) a population-level risk stratification overview to anticipate care needs (empanelment), 2) training for healthcare professionals in cardiovascular risk communication, 3) a lifestyle questionnaire completed by patients before consultation (Lifestylecheck) and 4) a clinical decision support tool (U-Prevent). The program aims to support a more targeted use of available resources by identifying patients at higher risk and tailoring preventive efforts to those who may benefit most, thereby potentially improving the efficiency and effectiveness of care. Patients between 40–90 years of age enrolled in integrated care programs (programs developed to ensure a continuum of care for patients with chronic conditions) for increased cardiovascular risk are included. The primary outcome is defined as the proportion of the participants with successful achievement of all protocol-defined cardiovascular preventive treatment goals at 18 months of follow-up. Secondary outcomes include implementation outcomes (penetration and fidelity), healthcare professionals' satisfaction on usability of the PROSPERA program, patient experience with shared decision making and decisional conflict when making a healthcare decision, and outcomes on cost-effectiveness.
**Ethics and dissemination** Consent for the use of routine healthcare data is obtained through an opt-out procedure for participating practices. Digital informed consent will be obtained for the study questionnaires for patients and healthcare professionals. Main findings on effectivity, efficacy and implementation will be disseminated via peer-reviewed journals and via

(inter)national conferences.
**Trial registration number** NCT06593704

**Comment 3: Definition of the intervention provider**

*The manuscript relies heavily on the term "healthcare professional" throughout the description of the intervention, but it remains unclear who is actually responsible for delivering the various components of the PROSPERA programme. While the roles of Dutch practice nurses are described and the manuscript notes that GPs retain overall clinical responsibility, the protocol does not clearly specify which professional groups are expected to:*

*- review empanelment lists,*
*- prioritise patient invitations,*
*- complete the training,*
*- conduct consultations using Lifestylecheck and U-Prevent,*

*and contribute to implementation assessments.*
*This is particularly important because the intervention appears to target clinician behaviour as much as patient care. A clearer description of the professional roles involved, and who is considered the primary intervention provider, would improve both understanding and reproducibility of the protocol.*

**Authors' response**

We've added a section (in red) on page 7 on different roles and responsibilities of the primary intervention provider (the healthcare professionals) during the PROSPERA study:

'Practice nurses are often the professionals that execute the consultations within the integrated care programs under GP supervision. Practice nurses independently provide primary care to specific groups of patients with chronic diseases. They focus on prevention, monitoring, patient education, and the provision of support and guidance to patients [20]. Practice nurses are generally employed by individual GPs or general practice groups. Before qualifying, practice nurses complete a one- or two-year education program. Practice nurses assume responsibilities from the GP; however, the GP retains final responsibility.

In the PROSPERA study, the healthcare professionals who routinely provide CVRM care-as-usual in their primary care practice will also be responsible for reviewing the empanelment lists every three months, prioritising patients for invitation and the consultations with patients using the Lifestylecheck and U-Prevent. This process may be conducted by either a GP or a practice nurse. A prerequisite for using the empanelment list to prioritise patients and conducting individual PROSPERA CVRM consultations is completion of the PROSPERA accredited training in cardiovascular risk communication and participation in the initiation visit.'

**Comment 4: Consequences of risk stratification**

*I understand how patients are assigned to risk groups, but I do not yet understand what different care those groups receive. Despite the additional explanation provided regarding empanelment, the protocol still does not clearly explain what patients in the different risk panels actually experience. The manuscript describes how patients are classified into normal, high, and extra-*

*high care demand groups, but it remains unclear what actions are expected to follow this classification. Specifically, readers would benefit from a clear description of:*
*- how management differs between the three panels;*
*- how panel assignment influences patient invitation, review frequency, follow-up, or treatment;*
*- whether specific actions are recommended for each category; and*
*- how much of the subsequent care pathway is standardised versus left to clinician discretion.*
*At present, the protocol clearly describes the risk stratification process but does not clearly describe the consequences of stratification. This makes it difficult to understand the active ingredients of the intervention and how they differ from usual care.*
*In summary, despite your substantial revisions, several issues remain unresolved with respect to the intervention - which is insufficiently specified and inaccessible to readers.*

**Authors' response**
We thank the reviewer for this comment. We would partially like to refer to our response provided for comment 1 and to the text below, which is a revision of the previous paragraphs on empanelment on page 10 (in red):

'Empanelment is implemented as a population-level tool for proactive care management and is conducted by the research team every three months. Participating primary care practices receive an overview of their integrated care population at risk for CVD, stratified according to expected CVRM care demand into three panels: normal, high, and extra-high. During the training and the initiation visit, the research team will guide healthcare professionals in interpreting and appropriately using this overview.

The purpose of the empanelment overview is to enable healthcare professionals to identify patients who might benefit from proactive CVRM management and to prioritise patients for invitation and follow-up. Thus, panel assignment does not constitute a treatment algorithm and does not prescribe a specific clinical intervention. Rather, it provides structured information to support decisions about which patients to prioritise, when to invite them for CVRM review, and how intensively to follow them.

Although the decision as how to act upon the population risk stratification remains with the healthcare professional, the intended implications of the three normal, high, and extra-hight panels were as follows. Patients in the **normal panel** generally required less proactive CVRM management when treatment goals were achieved and no relevant CVD history was present. These patients could therefore be reviewed at a lower frequency, with renewed CVRM assessment according to the usual clinical context and changes in health status. Patients in the **high panel** could be prioritised for more active CVRM management because of an increased need for risk-factor assessment and/or treatment optimisation. For these patients, healthcare professionals could use the individual-level PROSPERA components, including U-Prevent and the Lifestylecheck to support individual risk assessment, lifestyle assessment, and shared decision-making. Patients in the **extra-high panel**, characterised by a history of CVD and treatment goals not being achieved, could be prioritised for the most intensive proactive management and follow-up, with the aim of identifying unmet treatment needs and supporting treatment optimisation.'

**<u>Reviewer #3</u>**
Dr. Jennifer Ridgeway, Mayo Clinic

**General remarks of the reviewer**
*I thank the authors for their consideration of the prior comments. New details improve the transparency of the authors' work. The manuscript has remaining weaknesses, though. These are primarily related to implementation outcomes and triangulation of implementation and effectiveness data.*

> **General response of the author**
> We thank the reviewer for continuing to help us improve the manuscript. Please see below the comments addressed point-by-point and the adaptations made to the manuscript.

**Comment 1: rationale for a Hybrid Type 2 study**
*While studying implementation and effectiveness at the same time is indeed consistent with this design, the implementation strategies and outcomes are not well specified. How are strategies implemented and evaluated (given target implementation outcomes)? How does existing evidence inform your decision to call this a Hybrid Type 2 design?*

> **Authors' response**
> Thank you for your observations.

First, with respect to the implementation and evaluation of strategies we added the following (in red) to the manuscript on page 8 in the method section:

> 'An implementation ambassador will be appointed in each primary care practice participating in the intervention arm. This general practitioner or practice nurse serves as the primary point of contact for the researchers and is offered implementation strategies (e.g. training, user guides, pocket cards) for guiding the implementation of the intervention. At the start of the trial, we will conduct an initiation visit in each primary care practice in order to familiarise the healthcare professionals with the PROSPERA program. During this visit, we will systematically work through all components and all stages of the program, including all supporting materials.
>
> Other implementation strategies incorporated into the study include aligning the intervention with specific and frequently used EHRs, stimulating healthcare professionals' engagement with the program by distributing newsletters with tips and tricks, organising Q&A sessions, and improving the accessibility of the intervention by restructuring the digital- and physical environment in the consultation room (i.e. providing intervention components such as the Lifestylecheck in paper format or by placing a desktop shortcut to the automatically pre-filled U-Prevent medical device on the computer) during the initiation visits. Implementation strategies will be evaluated via the pre-defined implementation outcomes and during a final implementation gap visit at the end of the study.'

Second, regarding the definition of the present study as a hybrid type II study, we followed the framework by Curran et al. (2012, 2022). According to this framework, the distinction of a study between hybrid type I or hybrid type II is determined by the nature of the study outcomes and the extent to which implementation strategies are used. Curran et al. (2012) states that primary measures of implementation in a type II study should include the *'adoption of clinical treatment*

*(the PROSPERA intervention) and fidelity to it, as well as related factors.'* This is consistent with the terminology as proposed by Proctor et al. (2011) which will be used to define implementation outcomes. Although hybrid studies exist on a continuum as described by Curran et al. (2022), our study most closely aligns with a hybrid type II design, as it has a dual focus formulating research questions about both the effectiveness of the intervention as a whole and its implementation strategies.

We therefore made the following alterations (in red) to the manuscript on page 6:

> For the definition of the present study as a hybrid type II study, we followed the framework by Curran et al. (2012, 2022). According to this framework, hybrid studies exist on a continuum and the distinction of a study between hybrid type I or hybrid type II is determined by the nature of the study outcomes and the extent to which implementation strategies are used and evaluated. As our study has a dual focus formulating research questions about both the effectiveness of the intervention and its implementation strategies, the hybrid design of PROSPERA is classified as a type II study.

**Comment 2: description of triangulation**
*This would benefit from greater depth and explanation. How do implementation findings explain variation? What do the authors mean by triangulation in this case? How will they use fidelity data in effectiveness analyses?*

> **Authors' response**
> We agree with the reviewer that this concept deservers further elaboration. With triangulation we intent to assess whether an association exists between the effectiveness of the intervention, as measured by the primary outcome at the specific practice, and the degree to which the intervention was delivered as intended using implementations strategies. Although this will not explain variation in effectiveness causally, it could support its interpretation. Triangulation in this context refers to comparison of complementary data sources to understand why we will or will not find the PROSPERA program to be effective.

We have therefore added the following to the manuscript (in red) to the existing paragraph on page 13:

> 'Implementation outcomes will be used to better understand whether variation in fidelity and penetration across practices is related to clinical effectiveness variation [17, 34]. We will explore whether the effectiveness of the intervention, as measured by the primary outcome at the individual practice level, is associated with the extent to which the intervention was delivered as intended and which implementation strategies were used. These exploratory associations will not be interpreted causally, but are intended to explain heterogeneity in the observed outcomes. The evaluation of the implementation determinants and used strategies will also inform development of further implementation strategies aimed at facilitating the transition of the PROSPERA intervention from trial-based research setting to routine clinical practice in the future.'

**Comment 3: fidelity questionnaire**
*Now that I am able to assess the English version of the fidelity questionnaire, I am not convinced that it will answer questions about how the intervention was adapted for local context. As a*

*protocol paper, this is less a design comment as a comment that this information is not clearly presented. Likewise, the procedures for assessing this on a case-by-case basis are not clear.*

**Authors' response**
The fidelity scale is developed to assess the extent to which the PROSPERA components are *used as intended* on a primary care practice level. -

Heterogeneity in primary care practice organization across the Netherlands is, however, substantial. We therefore have to assess use as intended and possible deviations/adaptations during the course of the trial. For illustrative purposes, consider the following example: '*Some healthcare professionals have indicated that they will distribute the Lifestylecheck via email to their patients, while others provide the questionnaire on paper in the waiting room. As there is much inter-practice variation in primary care practice organization in the Netherlands, we allow the practices to exercise the freedom to adapt distribution of the Lifestylecheck fitting to the specific workflow and patient population of their primary care practice.*'

We therefore made the following changes (in red) to the manuscript on page 13: -

'As this study is a pragmatic RCT, the substantial heterogeneity in the organisational structure of primary care practices in the Netherlands makes it difficult to anticipate all contextual factors a priori. Therefore, the PROSPERA program is standardised across intervention practices, while allowing for local adaptations during the trial period. Such variation is not considered a protocol deviation, provided that the program's general principle of providing proactive and tailored care to meet the true needs of an individual patient is maintained. Fidelity to the program will be monitored through bi-monthly contact with the intervention ambassadors, during implementation gap visits and quantitively using the fidelity scale completed twice during the study by the healthcare professionals to assess whether the intervention is used as intended.'

## VERSION 3 - REVIEW

| | |
|---|---|
| **Reviewer** | **1** |
| **Name** | **Hespe, Charlotte** |
| **Affiliation** | **The University of Notre Dame Australia, School of Medicine** |
| **Date** | **06-Oct-2026** |
| **COI** | |

Thank you to the authors for their careful and comprehensive responses to my previous comments. The revised manuscript provides substantially greater clarity regarding the PROSPERA intervention, particularly the definition and practical application of empanelment, the professional roles involved in delivering the intervention, and the

intended consequences of assignment to the normal, high and extra-high care-demand panels.

The revised abstract is also clearer and more accessible to an international primary care readership. Importantly, the manuscript now makes explicit that empanelment is intended to support prioritisation, invitation and intensity of follow-up rather than function as a prescriptive treatment algorithm, while appropriately retaining clinician discretion within this pragmatic intervention.

I consider my substantive concerns to have been adequately addressed and believe the protocol is now suitable for publication. There remain a small number of minor editorial issues that could be addressed during final editing, including changing "a p-value of 5% will be considered clinically significant" to "a p-value <0.05 will be considered statistically significant", together with correction of minor typographical and grammatical errors.

I therefore recommend acceptance subject to minor editorial correction.

---

| | |
|---|---|
| **Reviewer** | **3** |
| **Name** | **Ridgeway, Jennifer** |
| **Affiliation** | **Mayo Clinic, Center for the Science of Health Care Delivery** |
| **Date** | **11-Sep-2026** |
| **COI** | |

---

I appreciate the additional revisions to this manuscript.
